# carba Nicotinamide Adenine Dinucleotide Phosphate: Robust Cofactor for Redox Biocatalysis

**DOI:** 10.1002/anie.202017027

**Published:** 2021-05-10

**Authors:** Ioannis Zachos, Manuel Döring, Georg Tafertshofer, Robert C. Simon, Volker Sieber

**Affiliations:** ^1^ Chair of Chemistry of Biogenic Resources Campus Straubing for Biotechnology and Sustainability Technical University of Munich Schulgasse 16 94315 Straubing Germany; ^2^ Synbiofoundry@TUM Technical University of Munich Schulgasse 22 94315 Straubing Germany; ^3^ Roche Diagnostics GmbH DOZCBE.-6164 Nonnenwald 2 82377 Penzberg Germany; ^4^ Catalytic Research Center Technical University of Munich Ernst-Otto-Fischer-Strasse 1 85748 Garching Germany; ^5^ School of Chemistry and Molecular Biosciences The University of Queensland 68 Copper Road St. Lucia 4072 Australia

**Keywords:** biocatalysis, biomimic, cNADP, oxidoreductases, regeneration system

## Abstract

Here we report a new robust nicotinamide dinucleotide phosphate cofactor analog (carba‐NADP^+^) and its acceptance by many enzymes in the class of oxidoreductases. Replacing one ribose oxygen with a methylene group of the natural NADP^+^ was found to enhance stability dramatically. Decomposition experiments at moderate and high temperatures with the cofactors showed a drastic increase in half‐life time at elevated temperatures since it significantly disfavors hydrolysis of the pyridinium‐N−glycoside bond. Overall, more than 27 different oxidoreductases were successfully tested, and a thorough analytical characterization and comparison is given. The cofactor carba‐NADP^+^ opens up the field of redox‐biocatalysis under harsh conditions.

## Graphical abstract

A robust cofactor analog (carba‐NADP^+^) is described here with a 33‐times longer half‐life time when the cofactor is heated up to 50 °C. Decomposition experiments on the reduced form at low pH values showed a drastic increase in stability. The cofactor is found to make a significant contribution to the field of redox‐biocatalysis under harsh conditions retaining most of its enzymatic activity.

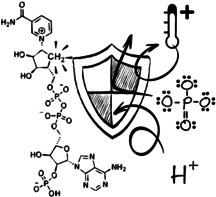

## Introduction

Biocatalysis has seen a major development in recent decades. The use of enzymes has become a method of choice in organic synthesis and various industrial applications.^[1]^ Thus, the enzyme market is growing. Until now, hydrolases have held the largest market share,^[2]^ but second place is occupied by oxidoreductases.^[3]^ This is not surprising since significant effort has been devoted to the field of biocatalytic oxidation as well as reduction reactions.^[4]^

Oxidoreductases are ubiquitous enzymes that catalyze redox reactions of a diverse range of chemicals with impressive specificity and selectivity. Among this widespread group, approximately 50 % are dependent on nicotinamide dinucleotide cofactors, namely NAD(H) or its phosphorylated equivalent NADP(H).^[5]^ Correlated with their different metabolic roles, NAD(P)^+^‐dependent oxidoreductases usually prefer one coenzyme as hydride (H^−^) transfer partner to the other. Reduction and oxidation of the substrate is achieved by binding both substrate and cofactor to the catalytic center, followed by transferring one hydride group between the substrate and the cofactor's nicotinamide moiety. It is worth mentioning that in some enzymes this can also happen indirectly via intermediate steps. Either way, for a complete conversion, stoichiometric amounts of expensive and metastable cofactor are required.

Necessitated by this, regeneration systems were developed in order to make biocatalysis, including redox enzymes, more cost‐effective.^[6]^ One of the most promising methods is still the enzymatic recycling of cofactors, due to their compatibility under similar reaction conditions with respect to temperature, as well as pH and ionic strength. But it is worth mentioning that chemical, electrochemical, and photocatalytic, as well as homogenous and heterogeneous catalytic approaches also exist.^[7]^

The number of biotransformations that take advantage of such recycling systems is increasing constantly, and interesting concepts have been reported for sustainable cell‐free multi‐enzyme cascades, taking advantage of enzymatic redox chemistry, producing biobased fuels and bulk chemicals,^[8]^ and even more so for fine chemicals.^[9]^ Either way, production costs need to be minimized, and because NADP(H) is less stable and more expensive than the non‐phosphorylated equivalent, switching the cofactor preference of oxidoreductases is an issue of general interest.

To facilitate this task, online tools such as CSR‐SALAD have been developed.^[10]^ Generally, switching the cofactor acceptance from NADP(H) to NAD(H) is more challenging than the other way around,^[11]^ although naturally many exceptions do exist to this rule.[^12^, ^13^] Nevertheless, cofactor stability and continuous supply remain a limitation, not only for cost‐effective production but also for ensuring steady diagnostic results under varying ambient conditions (moisture, temperature, etc.) but even more so for high‐temperature applications. And while enzyme engineering and tapping genetic diversity from extremophilic organisms have allowed the development of highly thermostable enzymes with high TTN at elevated temperatures, for the redox cofactors it was not possible to keep up.^[14]^

For this reason, one of the current challenges in biocatalysis is to develop and optimize simple, efficient, and, in particular, robust cofactors including their recycling systems. This can be achieved by developing either cheaper cofactors or more stable cofactors with higher total turnover numbers. There are a variety of nicotinamide biomimetic cofactors described in literature.^[15]^ Although many of them show notable properties, only a small group of them can be considered as stable per se.^[16]^ In addition, many of them, even if the redox moiety is similar, are not well accepted by most enzymes. Therefore, biocatalysts firstly need to be engineered. Recently, such an engineering study was performed by Black and co‐workers yielding a *Bacillus subtilis* glucose dehydrogenase accepting the semi‐synthetic cofactor nicotinamide mononucleotide (NMN^+^).^[17]^

As expected, this becomes even more difficult when we try to reduce fully synthetic nicotinamide cofactors (NCBs) using enzymes. To the best of our knowledge, only one glucose dehydrogenase from *S. solfataricus* thus far described is capable of reducing NCBs.^[18]^

On the contrary, remarkable results were achieved for oxidation reactions of such biomimetic cofactors using flavoenzymes,^[19]^ which, inter alia, result from altered redox potentials.^[16]^

**Scheme 1:**
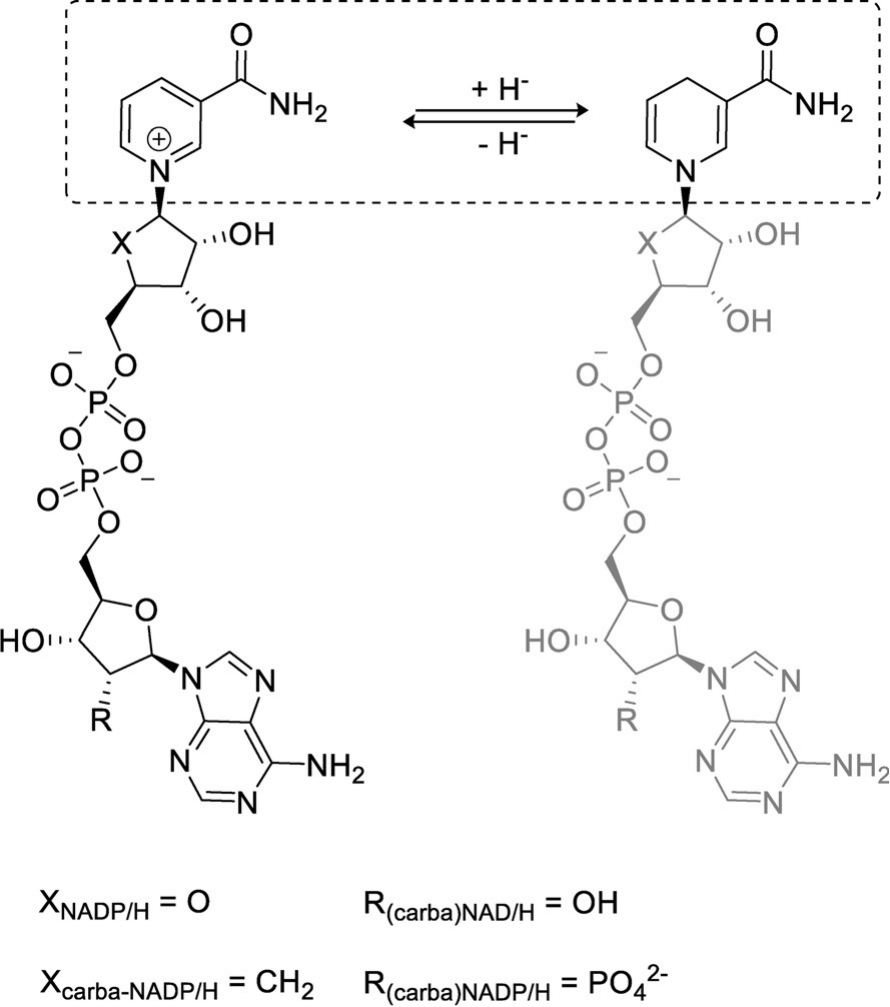
Structure of nicotinamide adenine dinucleotide cofactors; left: the oxidized cofactor (carba)‐NAD(P)^+^; right: the reduced form of (carba)‐NAD(P)^+^. In the artificial cofactor β‐d‐ribose oxygen (X) has been replaced by a methylene group resulting in a 2,3‐dihydroxy cyclopentane ring.

Yet all of these approaches do not readily address the issue of cofactor instability, which is attributable to the fact that nicotinamide‐based cofactors are prone to hydrolysis of the pyridinium‐N−glycoside bond.^[20]^ Structure elucidation of the thermal degradation products showed that hydrolysis and oxidative ring opening of the reduced nicotinamide adenine dinucleotide (phosphate) were indeed the main reaction paths.^[21]^ Further, the decomposition of NADP(H) depends on many diverse factors like temperature, pH,^[22]^ ionic strength, and also the presence of particular anions such as phosphate^[23]^ or acetate.^[24]^ It has been shown that carba‐NAD^+^, where the β‐d‐ribose of the nicotinamide ribonucleoside has been replaced with a 2,3‐dihydroxycyclopentane ring (Scheme 1), is a non‐hydrolyzable NAD^+^ derivative. Initially, this cofactor was designed in the late 1980s by Slama and Simmons to serve as a specific inhibitor to study the biological function of NAD‐glycohydrolases and ADP‐ribosyl transferases.^[25]^ Later reports showed an enzymatic reduction for the first time, using a glucose dehydrogenase plus an enzymatic synthesis route of carba‐NAD^+^ patented by Roche Diagnostics.^[26]^ carba‐NAD(H) turned out to be an advantageous alternative to NAD(P)/H for glucose dehydrogenase‐based glucose sensing and found its way towards an application.^[27]^ Interestingly, recent reports claim that the enzyme stability of glucose dehydrogenases increases significantly when stored together with the stable carba analog.^[28]^ In this study, we aimed to thoroughly analyze the general suitability of carba‐NADP^+^ for redox biocatalysis. Besides general information on stability, the new cofactor was characterized and compared with the natural one in terms of acceptance and activity with a large number of enzymes. Moreover, the enhanced stability is exploited and demonstrated in selected cases.

## Results and Discussion

**Figure 1:**
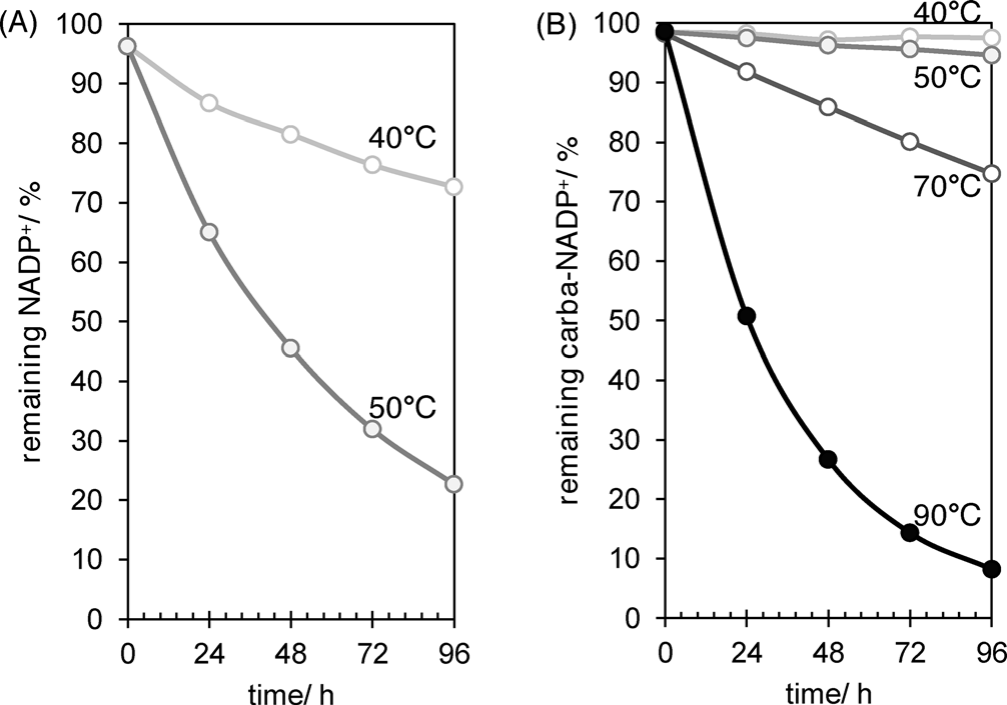
Thermal cofactor decomposition for A) NADP^+^ and B) carba‐NADP^+^ at different temperatures determined by achiral HPLC. A stock solution of the respective cofactor (10.0 mg mL^−1^) was prepared in potassium phosphate buffer (100 mm, pH 7.0) in an eppendorf vial and incubated in an eppendorf thermomix (600 rpm, horizontal position, 40–50 °C and 40–90 °C).

We first performed a comparative analysis of the half‐life time for both cofactors NADP^+^ (Figure 1 A) and carba‐NADP^+^ (Figure 1 B) at elevated temperatures. For this, the natural and non‐natural cofactors were incubated within a 100 mm KPi buffer at different pH values. At defined periods of time, samples were withdrawn and analyzed via HPLC (Figure S2). As expected, the NADP^+^ level is reduced by 80 % after 100 h at 50 °C, whereas carba‐NADP^+^ in contrast is barely affected. To test the limits of the carba cofactor we heated the system to nearly boiling point. But even at 90 °C, more than approx. 50 % are still intact after one day of incubation. The extrapolated half‐life time at 50 °C is more than 1200 h for the artificial cofactor, as compared to 40 h for the natural one (30 times more stable).

**Figure 2:**
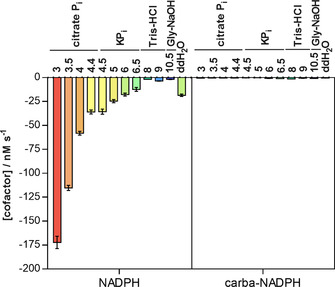
pH‐dependent cofactor decomposition for cofactors NADPH (left) and carba‐NADPH (right). Cofactors were incubated at 25 °C in different buffer systems: from left to right pH 3–4.4 citrate phosphate buffer (citrate P_i_), pH 4.5–6.5 potassium phosphate buffer (KP_i_), pH 8–9 Tris buffer (Tris‐HCl), pH 10.5 glycine buffer (Gly‐NaOH) each 100 mm if not stated otherwise and as control in pure water (ddH_2_O).

One limiting factor that we already addressed in the introduction is the issue of the reduced form not being stable at low pH. This may be of interest in particular applications that, for example, require processing steps in which a low pH is required. Thus, we were curious to know whether carba‐NADPH would also survive acidic treatment. A comparison of NADPH and carba‐NADPH shows that while the conventional cofactor decomposes at pH 3 at a rate of 175 nm s^−1^, the artificial one stays almost unaffected at a rate below 1 nm s^−1^ (Figure 2).

In order to ensure that the remaining cofactor was still intact for enzymatic conversion, it was enzymatically oxidized using the NADP(H) oxidase *Lp*NOX from the organism *Lactobacillus pentosus* and following absorption at 358–360 nm (Figure S15).

While the extraordinary stability of carba‐NADP^+^ is encouraging, the major question of whether carba‐NADP^+^ is accepted by different enzymes remains. Thus, we tested both commercially available biocatalysts and heterologously produced ones. In total, 52 different enzymes and variants were investigated using one or more substrates (Table S4). We were pleasantly surprised that most biocatalysts led to a successful conversion. Generally, we found that most enzymes that naturally accept NADP^+^ will also tolerate the carba modification. Focusing initially on cofactor‐recycling systems in general, we identified a collection of active alcohol‐, glucose‐, and glucose‐6‐phosphate dehydrogenases, which are commonly applied as recycling systems for the reduction of NADP^+^ (Entry 15–18, Table S4). Further to this, we also report a flavin‐dependent NAD(P)H oxidase as an important tool for the opposite recycling direction.

As expected, commercially available FDH did not accept carba‐NADP^+^ well, since it is strictly NAD^+^ dependent (Entry 45, Table S4). It is worth mentioning here that we found a slight activity in the lower milli‐unit range, but we would not consider it “active” in terms of usability. Due to a lack of proper production facilities, we also did not try out FDHs containing molybdenum–tungsten,^[29]^ or others. Finally, yet importantly within the group of regeneration systems, we successfully tested phosphite dehydrogenases (elsewhere phosphonate dehydrogenases) as the NADPH regeneration system.^[30]^ The thermodynamically favorable oxidation of phosphite to phosphate by phosphite dehydrogenase makes this class of enzyme useful for cofactor regeneration (Entry 35–36, Table S4).

To expand the repertoire of biocatalysts beyond regenerative enzymes, we continued investigating reductive aminases, imine and enoate reductases, lactate, aldehyde, α‐ketoglutaric semi‐aldehyde, succinic semialdehyde, aldonate/polyol and dihydrolipoyl dehydrogenases, as well as the P450 BM3 and Baeyer–Villiger monooxygenases (Entry 33–34, Table S4). Nearly all of the enzymes mentioned are well‐known examples that are frequently applied in biocatalysis, and many of them have already been improved in terms of thermostability.

In general, working at elevated or even high temperatures can be important in process optimization in order to overcome many limiting factors.^[31]^ One current example is the development of minimized biocatalytic reaction cascades in cell‐free processes for the conversion of glucose to ethanol or isobutanol using only six or eight enzymes, respectively. Enzymes required for such routes are being developed to work at elevated temperatures.^[32]^ The production of alcohols working at high temperatures can be useful to enable easy removal of the volatile product and reduce the risk of contamination. In other examples, it might be extremely useful for increasing solubility of several substrates and intermediates.

**Figure 3:**
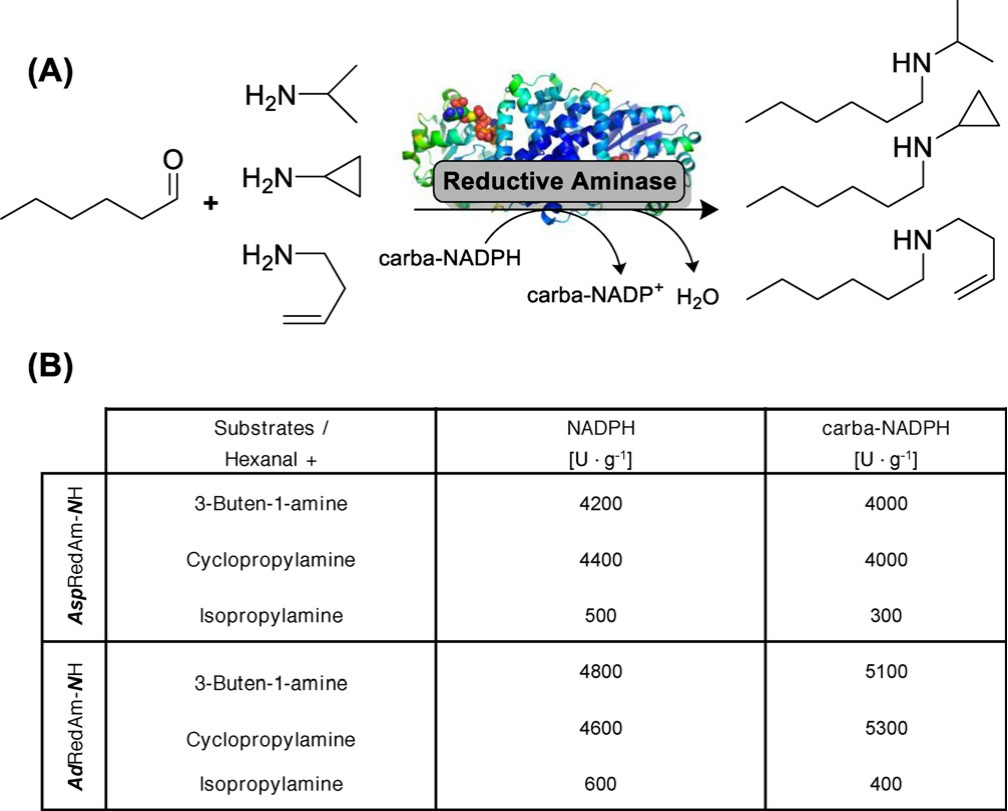
A) Reaction scheme for tested reductive aminases *Asp*RedAm and *Ad*RedAm converting hexanal and various amine sources utilizing carba‐NADPH. B) Activities with reductive aminases using NADPH or carba‐NADPH, hexanal, and various primary amines. Reaction conditions: 100 mm Tris‐HCl buffer pH 8.2, 5 mm hexanal, 10 mm amine, 1 mm cofactor, 0.2 mg mL^−1^ enzyme, 30 °C.

This might be attractive for the attention‐grabbing class of reductive aminases (RedAm) that are strictly NADPH dependent and catalyze the coupling of mostly aliphatic carbonyl compounds with a variety of primary amines (Figure 3 A). We tested the recently described *Asp*RedAm and also *Ad*RedAm since it is slightly more thermotolerant (Entry 27–28, Table S4). Both gave comparable results with the natural and carba cofactor. Interestingly, *Ad*RedAm showed a slight improvement (1.4‐fold activity) with carba‐NADPH over the natural one. Similar enzyme classes like the imine reductase IRED‐(*S*)‐*Pe* also ran fine with the reduction of 3,4‐dihydroisochinoline.

Equivalent activities were also measured for a set of flavin‐dependent enzymes comprising the self‐sufficient P450 BM3, two Baeyer–Villiger‐monoxygenases (CHMO and HAPMO) and an enoate reductase (*Ts*ER from *Thermus scotoductus*). Even if not always applicable literature shows that quite a number of flavin‐dependent oxidoreductases seem to be capable of utilizing a wide range of biomimetic cofactors. This is not too surprising as the *proS*‐hydride transfer from the nicotinamide to the flavin can be achieved efficiently through spatial proximity and does normally require fewer binding events with buried binding pockets to stabilize the transition state, meaning that hydride transfer, and not a structural change, determines the rate in this reductive half‐reaction.^[33]^

Besides the FDH mentioned above, further experiments were performed to test strictly NAD‐dependent enzymes including diaphorase^[34]^ as well as uronate, borneol, aldehyde, semialdehyde, glyceraldehyde‐3‐phosphate, *meso*‐2,3‐butanediol, glucose and alanine dehydrogenase (Entry 61, 53, 52, 46–47/55–56, 60, 57, 51, 50, and 58, respectively Table S4). As expected, we did not find any significant activity in those reactions.

In general, all enzymes that naturally accept NADP(H) will also accept carba‐NADP(H) with only a few exceptions. Oxidation of carba‐NADPH works extremely well, while reduction of carba‐NADP^+^ seems to be more difficult in nearly 30 % of all cases. Here activity is reduced by a factor of 2–10 depending on the enzyme and the substrate. However, this cannot be generalized since we also found that the substrate preference changes with the cofactor. It is noteworthy that the cofactor preference can probably be selectively engineered as different ADH variant activities imply (Entry 6–8, Table S4).

An exception to the rule of carba‐NADP^+^ acceptance is given by many members within the class of aldehyde dehydrogenases. This is probably owed to the special NAD(P)^+^ binding mode caused by a different Rossman fold type in many aldehyde dehydrogenases.^[35]^ For example, the recently published aldehyde dehydrogenase from the archaeon *Thermoplasma acidophilum* showed only 5 % residual activity in the oxidation of glyceraldehyde (50 U g^−1^ instead of 1000 U g^−1^). Using the same substrate with a succinic semialdehyde dehydrogenase from *Escherichia coli* resulted in 1.5 % residual activity (2000 U g^−1^ instead of 150 000 U g^−1^). Testing a handful of other aldehyde dehydrogenases mostly yielded similarly strongly reduced activities, with one exception: An aldehyde dehydrogenase from *Geobacillus stearothermophilus* that has nearly identical speed (2000 U g^−1^) for the oxidation of butanal to butyric acid (Figure S12). However, when the less accepted isobutanal is chosen as substrate (Figure S11), a discrepancy arises and carba‐NADP^+^ performs three times worse. Somewhat in between are the α‐ketoglutaric semialdehyde dehydrogenase from *Pseudomonas putida* to run at 40 % velocity under the tested conditions (7000 U g^−1^ instead of 18 000 U g^−1^).

**Figure 4:**
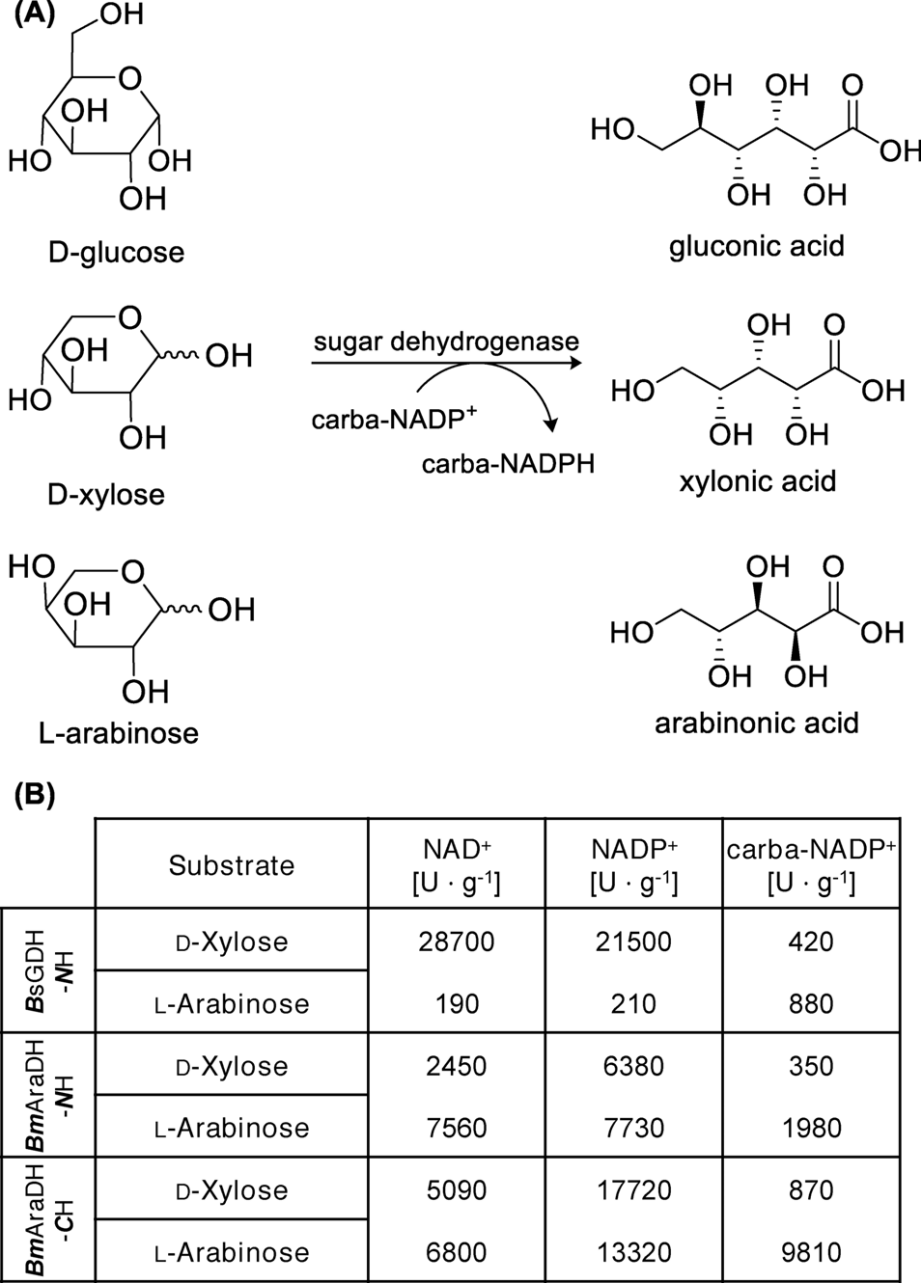
Analysis of sugar dehydrogenases from *Bacillus subtilis* and *Burkholderia multivorans*. A) Enzymes form sugar acids converting various sugar substrates like d‐glucose, d‐xylose, and l‐arabinose utilizing carba‐NADPH. B) Activities with sugar dehydrogenases using NADPH or carba‐NADPH. Influence of His‐tag on *Bm*AraDH (N‐terminal versus C‐terminal version). Reaction conditions 50 mm Tris‐HCl pH 8, 400 mm substrate, 500 μm cofactor, 30 °C.

Looking deeper into the kinetic parameters of a selection of five enzymes shows important features that come along with the new cofactor (Table S5). The first enzyme we analyzed is a promiscuous thermostable glucose dehydrogenase originating from the archaeon *Saccharolobus solfataricus* (*Ss*GDH). This enzyme was shown to act on a set on nicotinamide biomimetics.^[36]^ The activity of *Ss*GDH against d‐glucose is reduced by approx. 50 % with the artificial cofactor. However, this looks different when the sugars d‐xylose, l‐arabinose, and d‐galactose are converted (Figure S3–S6). Substrate inhibition, which occurs in the natural setup, seems to have completely vanished when carba‐NADP^+^ was chosen as cofactor and specific activities reached values that were up to six times higher (for d‐xylose 12 000 U g^−1^ instead of 2000 U g^−1^). We were also able to detect velocities that were three times higher in combination with the thermostable *Pfu*ADH for reduction and oxidation reaction at 65 °C, while *Bst*ADH, in contrast, performed 100 times worse in the oxidation of *n*‐butanol, but functioned reasonably well in the reduction reaction (1300 U g^−1^ instead of 1100 U g^−1^). Another interesting observation is the fact of a decreased Michaelis constant (*K*_m_) for the biomimetic for most of the enzymes, again except for aldehyde dehydrogenases that were analyzed. We have been wondering what could be the cause of carba‐NADP(H) showing such heterogeneous behavior towards different enzymes, and even more for different reaction directions in a single enzyme. It appears that there is no uniform pattern between different enzymes. Hence, we conclude that the kinetic changes are caused by the structural difference of both cofactors. In silico calculations showed a different conformation of the two structures after energy minimization (cf. in silico part). Bearing in mind that even small sub‐Ångström shifts of reaction partners can have drastic effects on reaction parameters, we think that the rotation of the nicotinamide and adenine moiety consequently changes energy levels and thus the binding event during catalysis. Consequently, different substrates may form altered interaction patterns with the cofactor. This is also the case for the *Bs*GDH which shows 50‐fold reduced activity with carba‐NADP^+^ compared to NADP^+^ when d‐xylose is used as substrate. However, when using the less accepted substrate l‐arabinose activity increased by a factor of four (Figure 4).

It is well known that affinity‐tag additions may result in altered enzymatic function and protein parameters.^[37]^ Hence, we also compared one His‐tagged dehydrogenase as N‐terminal and C‐terminal version, respectively. For *Bm*AraDH with N‐ and C‐terminal His‐tag, activity towards the less‐accepted substrate d‐xylose has decreased almost 20‐fold. This is different for l‐arabinose. While for the *N*‐His version activity drops to approx. 2 U mg^−1^, interestingly almost 10 U mg^−1^ remain for the *C*‐His construct. Apparently, the position of the His‐tag influences the acceptance of the different nicotinamide cofactors unevenly strongly, which might be attributed to very slight conformational changes of the protein in the presence of the tag.

Depending on each individual binding site architecture and co‐substrate these parameters will differ. Consequently, e.g., *Ss*GDH loses more than 50 % of its initial activity for d‐glucose but gains activity for the somewhat smaller substrate d‐xylose. Protein structure determination including carba cofactors as ligands could give valuable information on precise binding modes.

**Figure 5:**
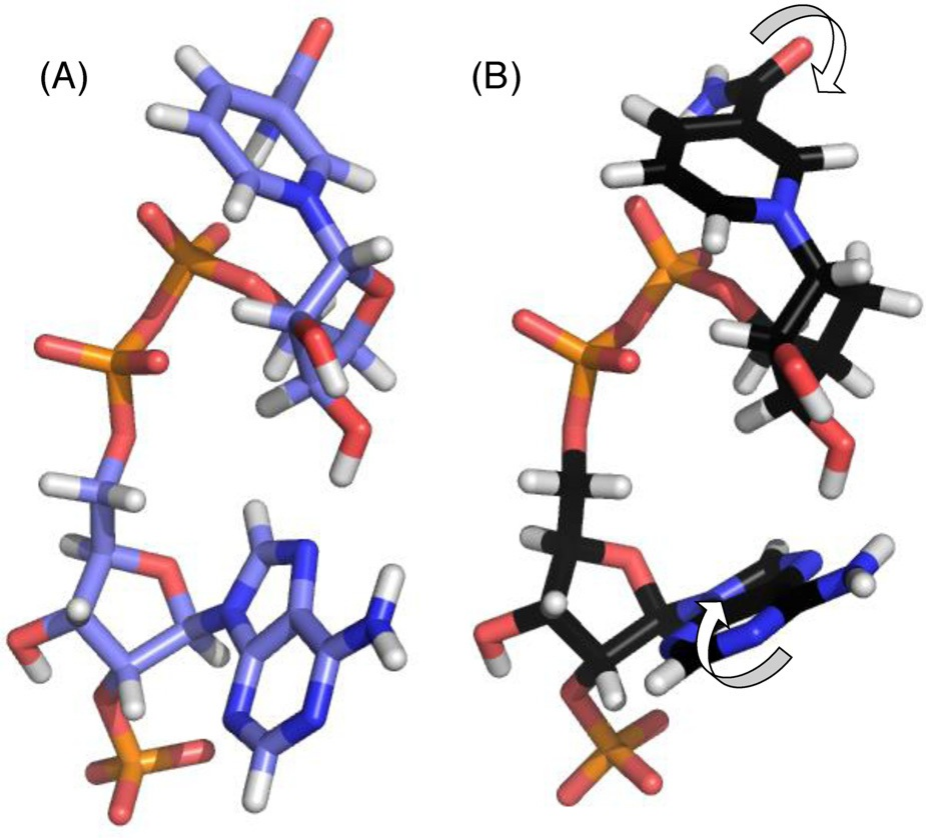
3D cofactor structures after energy minimization in Yasara using Amber99 force field. A) NADP^+^ and B) carba‐NADP^+^ after energy minimization, shows rotation of the nicotinamide and adenine moiety (arrows). Reduced cofactor states can be found in Figure S17.

Taking a close look at the NAD(P)^+^‐binding domain of glucose‐6‐phosphate dehydrogenases from *Saccharomyces cerevisiae* and *Leuconostoc mesenteroides* (Figure S18), we can see that the pocket volume is reduced in *Sc*G6PDH at position Y95 compared to V86 in *Lm*G6PDH. Thus, it is harder to realize conformational changes to the carba‐NADP^+^ adenine moiety (Figure 5) in the *Saccharomyces* enzyme. This is also found in the residual activities, which make up only 5 % for *Sc*G6PDH but 30 % for *Lm*G6PDH. Nevertheless, overall sequence identity is only 35 % and other effects surely will also affect ligand binding parameters.

## Conclusion

A key challenge in redox biocatalysis remains the supply of cofactors and the inherent stability of both the cofactor and the catalyst.^[38]^ Our comprehensive study on the new cofactor with various types of oxidoreductases proves that most enzymes that accept NADP(H) also accept carba‐NADP(H). Even though activity for some enzymes seems to be reduced, this is a good starting point for enzyme engineering and future studies will aim for engineering enzymes for higher carba‐NADP^+^ specificity and activity. However, we also found that some biocatalytic conversions work even more effectively with carba‐NADP(H). In addition, we showed that this artificial cofactor can serve as a very valuable tool in processes that require harsh conditions like extreme temperatures and pH and normally suffer from loss of expensive cofactors. The combination of carba‐NADP(H) with robust enzymes not only opens up the field of sole enzymatic redox chemistry at high temperatures, but could be of special interest for many chemo‐enzymatic approaches where harsh conditions are necessary.^[39]^

## Conflict of interest

This work resulted from non‐financial collaboration. The cofactor carba‐NADP (free acid) was provided by Robert C. Simon and Georg Tafertshofer (Roche Diagnostics).

## Supporting information

As a service to our authors and readers, this journal provides supporting information supplied by the authors. Such materials are peer reviewed and may be re‐organized for online delivery, but are not copy‐edited or typeset. Technical support issues arising from supporting information (other than missing files) should be addressed to the authors.

Supplementary
