# Supplementary material for: carba Nicotinamide Adenine Dinucleotide Phosphate: Robust Cofactor for Redox Biocatalysis
Source: Angew Chem Int Ed Engl. 2021 May 10;60(26):14701–6. doi: 10.1002/anie.202017027 (PMC8252718; doi:10.1002/anie.202017027)
Supplement: Supplementary file 1 — Supplementary [file ANIE-60-14701-s001.pdf]

Angewandte Chemie
Eine Zeitschrift der Gesellschaft Deutscher Chemiker

Supporting Information

carba Nicotinamide Adenine Dinucleotide Phosphate: Robust Cofactor for Redox Biocatalysis

*Ioannis Zachos, Manuel Döring, Georg Tafertshofer, Robert C. Simon, and Volker Sieber**

anie_202017027_sm_miscellaneous_information.pdf

Author Contributions

G.T. and R.C.S. performed production and temperature-dependent decay experiments on the cofactor. I.Z. and V.S. conceived the research on further stability experiments plus the enzymatical part. I.Z. performed experiments on the enzymatic acceptance as well as in silico studies. M.D. established a uniform kinetic measuring platform and evaluated kinetic measurements. All authors analyzed the data and worked on the manuscript.

Table of Contents

Table of Contents 1
Materials and Reagents 2
Standard Experimental Procedures 3
    Heterologous production of oxidoreductases 3
    Preparation of buffers for Äkta purifier 3
    Cell disruption by sonication 3
    His-tag proteins purification via IMAC 3
    Size exclusion chromatography (SEC) 3
    Determination of enzyme concentration 3
    Measurement of kinetic constants 4
    Enzymatic activity measurements for $NADP^+$ and carba-$NADP^+$ 5
    Production of NADPH and carba-NADPH 5
In silico section 5
Energy minimization of cofactors 5
Results and analytic data section 6
    Determination of water content in cofactor material 6
Hydrolysis profile of $NADP^+$ and carba-$NADP^+$ 7
Kinetic Measurements 8
Comparison of kinetic constants 17
Spectral analysis of (carba)-NADPH 18
3D modeling of nicotinamide cofactors 19
G6PDH structural differences 19
References 20
Author Contributions 20

Materials and Reagents

Table S1 Materials used in this study for kinetic measurements

Abbreviation	Labware	Brand	Order #
MTP-96	96-Well micro titer plate	Greiner bio one	655101
DWP	96-Well deep well plate	Greiner bio one	780271
lid	Lid for MTP	Sarstedt	82.1584.500
-	2 mL reaction tube	Eppendorf	-
LHS	Liquid handling station	Brand	-
-	Reservoir 6 x 40 mL	Brand	701456
50 µL tips	Pipetting tips 50 µL	Brand	732026
200 µL tips	Pipetting tips 200 µL	Brand	732028
1000 µL tips	Pipetting tips 1000 µL	Brand	732032
MCP	Multi-channel pipette (8 x 1000 µL, electric with multi dispense mode)	Eppendorf	
foil	Aluminum sealing foil	corning / axygen	PCR-AS-200

Table S2 Chemicals used in this study

Reagent	Distributer	Order #
(-)-Borneol	Sigma Aldrich	139114
(4-Hydroxyphenyl)ethan-1-one	Sigma Aldrich	278564
(*R*)-3-Hydroxybutyrate	Sigma Aldrich	54920-1G-F
(*S*)-3-Hydroxybutyrate	Sigma Aldrich	54925-1G-F
(*S*)-Lactaldehyde	Sigma Aldrich	47014
2-Butanol	Sigma Aldrich	19440
3,4-Dihydroisoquinoline	Merck	SAFA779385
3-Buten-1-amine (>97%)	Alfa Aesar	L20157.03
Benzaldehyde	Sigma Aldrich	12010-250ML-F
Butanal	Acros Organics	108091000
carba-$NADP^+$ free acid	Roche	-
Cyclohexanone	Sigma Aldrich	29140
Cyclopropylamine (99%)	Acros Organics	ACRO154880100
D-Galactose	Serva	22020.02
D-Glucose	Merck	108.337
D-Glucose-6-phosphate-disodiumsalt-dihydrate	Sigma Aldrich	67250
DMSO	Carl Roth	AE02.1
D-Xylose	Fluka	95731
Ethanol absolute	VWR	20.821.321
Glyceraldehyde	Sigma Aldrich	G5001
Glyceraldeyhde-3-Phosphate	Sigma Aldrich	G5251
HCl	Carl Roth	4625.1
Hexanal	Sigma Aldrich	115606
Isobutanal	Sigma Aldrich	418110
Isopropanol	Carl Roth	7343.1
Isopropylamine	Alfa Aesar	A15044.AE
L-Arabinose	Calbiochem	178680
L-Glutamate	Sigma Aldrich	1446600
Meso 2,3-Butanediol	Sigma Aldrich	361461
Na_2HPO_4	Carl Roth	P030.3
NAD^+	Carl Roth	AE11.2
NADH disodium salt	Carl Roth	AE12.2
$NADP^+$ disodium salt	Carl Roth	AE13.3
NADPH tetrasodium salt	Carl Roth	AE14.2
NaOH	Carl Roth	6771.2
n-Butanol	VWR	1.019.881.000
Octanoic acid	Carl Roth	2613
o-Phthaldialdehyde	Sigma Aldrich	P0657
Sodium phosphite dibasic pentahydrate	Acros Organics	ACRO428502500
Propionaldehyde	Sigma Aldrich	538124
Sodium pyruvate	Sigma Aldrich	P2256
Ammonium formate	Sigma Aldrich	17843
Succinic semialdehyde	Sigma Aldrich	14075
Tris HCl	Carl Roth	9090.3
Water (microfiltered)	Elga	microfiltered

Standard Experimental Procedures

Heterologous production of oxidoreductases

Some enzymes in this study, except commercially available enzymes (HlADH, LDH, FDH, G6PDHs, L-GluDH), were expressed using auto induction (AI) medium.[1] A single colony of *E. coli* BL21 (DE3) bearing plasmids with a gene of interest was grown overnight in a 100 mL baffled flask containing 10 mL LB medium supplemented with 100 µg/mL kanamycin or carbenicillin at 37 °C, 150 rpm. The following day, the 10 mL culture was transferred to a 5 L baffled flask containing 1 L AI medium supplemented with 100 µg/mL kanamycin or carbenicillin. The culture was shaken at 120 rpm. Different growth temperatures were used, depending on the respective enzymes being expressed. Thermotolerant enzymes (*Ss*GDH, *Pfu*ADH, *Sto*ADH, *Gst*ADH) were produced at 30 °C, mesophilic enzymes at 18 °C. At the end of protein expression, cells were pelleted by centrifugation at 4,000 x *g* for 15 min. The cell pellets were stored at - 80 °C prior to enzyme purification. For production of the flavin-dependent enzymes TsER, BM3, CHMO and HAPMO, TB media and the respective selection marker was used. For induction, the TB medium was supplemented with 1 mM IPTG once the OD at 600 nm reached a value of 0.8 - 1.

Preparation of buffers for Äkta purifier

Binding buffer: 50 mM KPi, 20 mM imidazole, 0.5 M NaCl, 10 % (w/v) glycerol in ddH$_2$O, adjusted with HCl to pH 8. The solution was filtered through 0.45 µm filter paper prior to use.
Elution buffer: 50 mM KPi, 500 mM imidazole, 0.5 M NaCl, 10 % (w/v) glycerol in ddH$_2$O, adjusted with HCl to pH 8. The solution was filtered through 0.45 µm filter paper prior to use.
Desalting buffer: KPi, and Tris buffer with different concentration and pH were used throughout this study.

Cell disruption by sonication

All cell disruption in this study was performed by sonication. Cell pellet [10 % to 20 % (w/v)] was dissolved in the binding buffer. DNAse 5 µg/mL and 1 mM MgCl$_2$ were added to the solution. The cell suspension in a 50 mL falcon tube was placed in ice and a sonotrode was inserted in the cell suspension. Sonication was performed for 20 min (0.5 s cycle and 80 %). Cell suspension was centrifuged at 20.000 x *g* for 30 min at room temperature. It is worth mentioning that SsGDH and *Pfu*ADH were heat treated in a water bath at 70 °C for 30 min before centrifugation. The supernatant was filtered through 0.45 µm filter before being applied on an Äkta purifier.

His-tag proteins purification via IMAC

Some Enzymes used in this study bearing N- or C-terminal hexa-histidine tag (cf. Table 1). Immobilized Metal Affinity Chromatography (IMAC) was used to purify all enzymes. Purification was performed using Äkta purifier (GE Healthcare). The protein supernatant was loaded on a 5 mL HisTrap FF crude column with 10 mL · min^{-1} flow rate. The column was washed with the binding buffer until the online UV detector at 280 nm showed absorption < 50 mAU (approximately 25 mL of the binding buffer) using 7 mL · min^{-1} flow rate. The principle of a His-tag purification is immobilized nickel ions in a His-tag column will interact with six histidines located at the terminal of the protein of interest, thus retained in a column. Other *E. coli* proteins would be washed away from the column during the washing step. To elute the enzyme of interest, the elution buffer was used with a 7 mL · min^{-1} flow rate. A higher concentration of imidazole will break the interaction between the nickel ions and the hexahistidine-tag thus allowing a protein of interest to be eluted from the column. A single fraction was collected. The column was equilibrated again with the binding buffer using a flow rate of 10 mL · min^{-1}.

Size exclusion chromatography (SEC)

SEC was used to remove imidazole and salt in the elution buffer. A 50 mL desalting column was applied to the Äkta purifier. Depending on the enzymes, a buffer of interest (desalting buffer) was used to equilibrate the HiTrap Desalting HiPrep 26/10 Desalting Column (GE Healthcare). Approximately 100 mL of a desalting buffer would be needed. A protein eluate from the previous IMAC purification was loaded to the desalting column. Maximum volume of 15 mL could be loaded to allow good separation. Upon loading, the desalting column was loaded to the column at a 10 mL · min^{-1} flow rate. A single fraction of protein in a desired desalting buffer was collected. The column was equilibrated with 100 mL of desalting buffer and made ready for the next protein elution. Salts and buffer of the elution buffer would be removed from the column during column equilibration.

Determination of enzyme concentration

Enzyme concentrations were determined using either Bradford assay (BioRad) or by UV-Vis Spectroscopy using extinction coefficients calculated by ExPAsy ProtParam tool.

Measurement of kinetic constants

An LHS (Liquid Handling Station, Brand) was used to increase the pipetting reproducibility and speed. For each kinetic, 96 separate reaction mixtures of the given composition were prepared in the wells of an MTP. Either the substrate solutions varied in concentration [S] or the cofactors [$NADP^+$] or [$cNADP^+$]:
12 differently concentrated substrate solutions [S] were placed in the upper half of the MTP, each time with the highest corresponding concentration of either [$NADP^+$] or [$cNADP^+$] (ranging from 0.5 mM – 5 mM, respectively) and each reagent mixture in duplicates:
12 substrate concentrations x 2 cofactors x 2 (duplicates) = 48 reaction mixtures
In the lower half of the MTP, 12 differently concentrated solutions of [$NADP^+$] or [$cNADP^+$] were added to the highest corresponding concentration of substrate [S] , also in duplicates:
12 cofactor concentrations of both cofactors ([$NADP^+$] or [$cNADP^+$]) x 2 (duplicates) = 48 reaction mixtures
The enzyme concentration [E] as well as the buffer concentration [buffer] and the measurement conditions (such as temperature of measurement ($T_{measure}$); volume (V), preheating time (t_{heat}), sequence of process steps (pipetting, preheating, addition of enzyme and measurement) were kept constant for the measurements of a kinetic. The reaction mixtures were pipetted in MTPs with the help of LHS, followed by preheating of the mixture and addition of 25 µL of enzyme solution was added with an electric MCP in multi-dispense mode, and measurement in an Epoch 2 - BioTek photometer. The following figure shows the 96-well kinetic plate, a combination of the substrate master plate and the cofactor master plate (Figure S1). Detailed values of concentrations and reaction conditions can be found in the corresponding figures and their discriptons.

Kinetics were analyzed and plotted using the respective equation.

Equation (I) Michaelis-Menten:

$$Y = \frac{v_{max} \cdot X}{K_m + X}$$

Equation (II) substrate inhibition:

$$Y = \frac{v_{max} \cdot X}{[K_m + X \cdot (1 + \frac{X}{K_i})]}$$

96 well kinetic plate

	1	2	3	4	5	6	7	8	9	10	11	12
**A**	[NADP+] max<br>ohne [S]	[NADP+] max<br>0,0004 x [S] max	[NADP+] max<br>0,001x [S] max	[NADP+] max<br>0,002 x [S] max	[NADP+] max<br>0,004 x [S] max	[NADP+] max<br>0,01x [S] max	[NADP+] max<br>0,01x [S] max	[NADP+] max<br>0,04 x [S] max	[NADP+] max<br>0,1x [S] max	[NADP+] max<br>0,2 x [S] max	[NADP+] max<br>0,4 x [S] max	[NADP+] max<br>[S] max
**B**	[cNADP+] max<br>ohne [S]	[cNADP+] max<br>0,0004 x [S] max	[cNADP+] max<br>0,001x [S] max	[cNADP+] max<br>0,002 x [S] max	[cNADP+] max<br>0,004 x [S] max	[cNADP+] max<br>0,01x [S] max	[cNADP+] max<br>0,01x [S] max	[cNADP+] max<br>0,04 x [S] max	[cNADP+] max<br>0,1x [S] max	[cNADP+] max<br>0,2 x [S] max	[cNADP+] max<br>0,4 x [S] max	[cNADP+] max<br>[S] max
**C**	[NADP+] max<br>ohne [S]	[NADP+] max<br>0,0004 x [S] max	[NADP+] max<br>0,001x [S] max	[NADP+] max<br>0,002 x [S] max	[NADP+] max<br>0,004 x [S] max	[NADP+] max<br>0,01x [S] max	[NADP+] max<br>0,01x [S] max	[NADP+] max<br>0,04 x [S] max	[NADP+] max<br>0,1x [S] max	[NADP+] max<br>0,2 x [S] max	[NADP+] max<br>0,4 x [S] max	[NADP+] max<br>[S] max
**D**	[cNADP+] max<br>ohne [S]	[cNADP+] max<br>0,0004 x [S] max	[cNADP+] max<br>0,001x [S] max	[cNADP+] max<br>0,002 x [S] max	[cNADP+] max<br>0,004 x [S] max	[cNADP+] max<br>0,01x [S] max	[cNADP+] max<br>0,01x [S] max	[cNADP+] max<br>0,04 x [S] max	[cNADP+] max<br>0,1x [S] max	[cNADP+] max<br>0,2 x [S] max	[cNADP+] max<br>0,4 x [S] max	[cNADP+] max<br>[S] max
**E**	0 [NADP+]max<br>[S] max	0,005 [NADP+]max<br>[S] max	0,075 [NADP+]max<br>[S] max	0,01 [NADP+]max<br>[S] max	0,015 [NADP+]max<br>[S] max	0,02 [NADP+]max<br>[S] max	0,03 [NADP+]max<br>[S] max	0,05 [NADP+]max<br>[S] max	0,07 [NADP+]max<br>[S] max	0,1 [NADP+]max<br>[S] max	0,5 [NADP+]max<br>[S] max	[NADP+]max<br>[S] max
**F**	0 [cNADP+]ma<br>[S] max	0,005 [cNADP+]ma<br>[S] max	0,075 [cNADP+]ma<br>[S] max	0,01 [cNADP+]ma<br>[S] max	0,015 [cNADP+]ma<br>[S] max	0,02 [cNADP+]ma<br>[S] max	0,03 [cNADP+]ma<br>[S] max	0,05 [cNADP+]ma<br>[S] max	0,07 [cNADP+]ma<br>[S] max	0,1 [cNADP+]ma<br>[S] max	0,5 [cNADP+]ma<br>[S] max	[cNADP+]ma x<br>[S] max
**G**	0 [NADP+]max<br>[S] max	0,005 [NADP+]max<br>[S] max	0,075 [NADP+]max<br>[S] max	0,01 [NADP+]max<br>[S] max	0,015 [NADP+]max<br>[S] max	0,02 [NADP+]max<br>[S] max	0,03 [NADP+]max<br>[S] max	0,05 [NADP+]max<br>[S] max	0,07 [NADP+]max<br>[S] max	0,1 [NADP+]max<br>[S] max	0,5 [NADP+]max<br>[S] max	[NADP+]max<br>[S] max
**H**	0 [cNADP+]ma<br>[S] max	0,005 [cNADP+]ma<br>[S] max	0,075 [cNADP+]ma<br>[S] max	0,01 [cNADP+]ma<br>[S] max	0,015 [cNADP+]ma<br>[S] max	0,02 [cNADP+]ma<br>[S] max	0,03 [cNADP+]ma<br>[S] max	0,05 [cNADP+]ma<br>[S] max	0,07 [cNADP+]ma<br>[S] max	0,1 [cNADP+]ma<br>[S] max	0,5 [cNADP+]ma<br>[S] max	[cNADP+]ma x<br>[S] max

Figure S1 Pipetting scheme of enzyme kinetics. Row **A + C** variation of $NADP^+$ with highest substrate concentration. **B + D** variation of carba-$NADP^+$ with highest substrate concentration. **E + G** variation of substrate with highest $NADP^+$ concentration. **F + H** variation of substrate with highest carba-$NADP^+$ concentration. Numbers given show relative (in comparison to the used substrate or cofactor-stock solutions) substrate and cofactor concentration in the kinetic plate. Colors do not correspond to any specific attribute.

Enzymatic activity measurements for NADP+ and carba-NADP+

All assays were performed in 96-well MTP plates in a reaction volume of 200 µL. Reactions were started by adding the enzyme.
A detailed overview is given in (Table 2) showing the preferred reaction, the acceptance towards both cofactors (NADP(H) and carba-NADP(H)) and further reaction conditions. For all reactions, multiple negative reactions, were performed without adding a cofactor, enzyme or substrate, respectively.

Production of carba-NADP+

The cofactor carba-NADP+ was prepared according to literature(WO 2011/012270 A1) by means of chemical and biocatalytical methods.[2]

Production of NADPH and carba-NADPH

10 mM of NADP+ and carba-NADP+ were fully reduced, each using 10 units of *Ss*GDH (glucose dehydrogenase heat-treated 30 min at 70 °C to degrade putative-bound cofactor) and 30 mM of D-glucose in a 20 mM Tris-HCl buffer at pH 8 at 37 °C. Reaction was followed spectrophotometrically. After full reduction, *Ss*GDH was removed using a centrifugal filter with 10 kDa cut-off. The reduced cofactor was stored at -80 °C until further use. 10 mM stock was diluted 1:10 within the final reaction mixture. Biocatalytic approaches using (carba)-NADPH thus contained 2 mM glucose, 1 mM gluconic acid and 0.2 mM Tris-HCl as contaminants.

In silico section

Energy minimization of cofactors

NADP+ cofactor structure was extracted from pdb: 2cdc (NADP from Molecule A). Structure was minimized in a second step. To get carba-NADP+ the respective ribose oxygen was swapped for carbon, and hydrogens were added. Cofactors were again minimized within the NOVA force field. The rotation of bonds was observed to be reversible when the carbon was swapped back to oxygen. It is worth noting that a water box around the cofactor (10 Å around all atoms, pKa neutralization to pH 7.4 and 0.9 % NaCl) suppresses most of the torsion.

Results and analytic data section

Determination of water content in cofactor material

Water content was determined using Karl-Fischer Titration to ensure comparable cofactor material. The water content of carba-NADP$^+$ was determined to be 8.7 % (w/w). The NADP$^+$ that was used contained similar amounts of water 6.2 % (w/w).

Table S3 Kar-Fischer titration data for determining cofactor water content is shown below.

Water standard: Hydranal water std 10,0 mg/g	
Lot#	SZBF1410V

	Sample			Measurement				
Measurement #	Syringe tara (g)	After addition (g)	Sample (g)	Addition titrant (mL)	µA	Titer		
1	6.9633	6.20157	0.76173	1.515	22	5.059	MW	5.0676
2	6.20173	5.58129	0.62044	1.229	26.1	5.0786	SD	-
3	5.58147	4.63693	0.94454	1.876	22.2	5.0676	Rel.std deviation	0.20%

	Sample			Measurement		
Measurement #	Syringe tara (g)	After addition (g)	Sample (g)	Addition titrant (mL)	µA	%H_2O
cNADP$^+$ (5g)	0.04472	0	0.04472	0.782	28.2	**8.862**
cNADP$^+$ (5g)	0.0065	0	0.0065	0.115	53.3	**8.966**
cNADP$^+$ (2g)	0.00598	0.00008	0.0059	0.097	28.3	**8.331**
NADP$^+$	0.00962	0.00033	0.00929	0.115	27.1	**6.273**
NADP$^+$	0.00659	0.00012	0.00647	0.08	21.2	**6.266**

Hydrolysis profile of $NADP^+$ and carba-$NADP^+$

To monitor the decomposition of carba-$NADP^+$ in comparison to $NADP^+$ HPLC, measurements were performed after a defined period of incubation at 50 °C - 90 °C. The decomposition of cofactors $NADP^+$ (disodium salt) and carba-NADP (free acid) was determined as function of time, temperature and pH-value: A stock solution of the respective cofactor (10.0 mg/mL) was prepared in a potassium phosphate buffer (100 mM, pH 6.0, 7.0 and 8.0, respectively) in an eppendorf vial and incubated in an eppendorf thermomix (600 rpm, horizontal position, 40 °C - 50 °C). After defined periods of time (24 hours), aliquots were withdrawn (25 µL), diluted with ddH_2O to reach a total volume of 1.00 mL.
The samples were measured on achiral HPLC afterwards. Column = RP18 ProntoSIL [150 mm × 4.6 mm], 5 µm dp [Bischoff]; flow rate = 1.0 mL/min; detection = 254 nm; oven temperature was set to 25 °C; HPLC program [buffer A: 20 mM KH_2PO_4, pH 6.5; buffer B: 20 mM KH2PO4, pH 6.5, 20 vol% MeCN] = 5.00 min 100% A, then over the next 5.00 min a gradient to 50% B; hold for 5 minutes 50% B, then go back to 100% A; total run time = 20.0 minutes. R_t ($NADP^+$) = 6.20 min, R_t (NAD^+) = 9.93 min; Rt (carba-$NADP^+$) = 3.33 min, R_t (c-NAD) = 9.42 min.

While $NADP^+$ (Figure S2 A) shows the formation of multiple degradation products, carba-$NADP^+$ (Figure S2 B) stays stable. Hence, no degradation products can be detected.

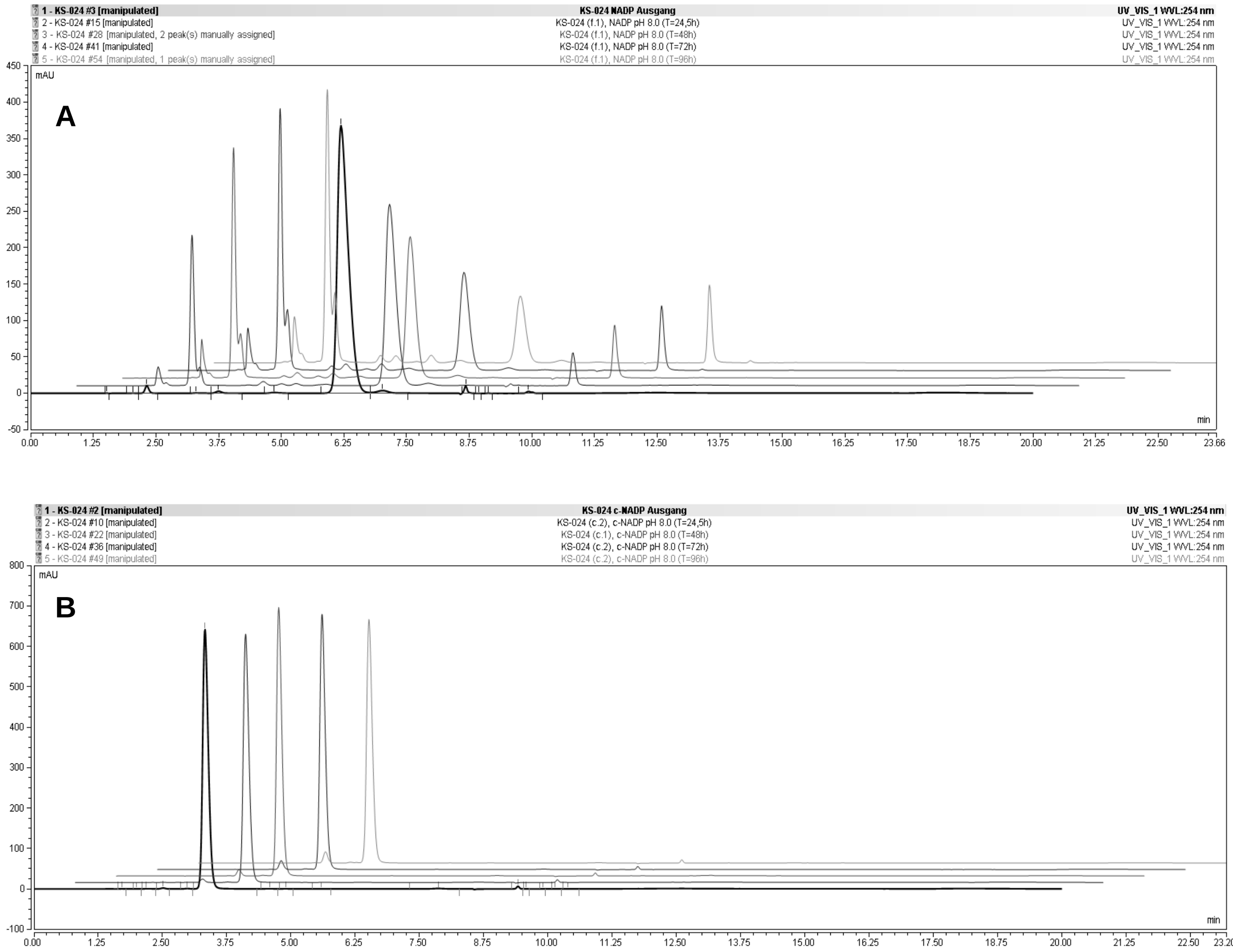

Figure S2 Hydrolysis profile of carba-$NADP^+$ (cNADP) Overlaid HPLC profiles of decomposition of **(A)** $NADP^+$ (50 °C, pH 8.0 in a 100 mM potassium phosphate buffer) over time (96 hours) **(B)** carba-$NADP^+$ (50 °C, pH 8.0 in a 100 mM potassium phosphate buffer) over time (96 hours).

Enzymatic acceptance of carba-NADP$^+$

In the following, a screening of several enzymes and their variants has been performed using 1 mM of cofactor. The substrate and reaction conditions stated in the respective column.

Table S4 List of biocatalytic reactions for comparative analysis of (carba)-NADP(H). Color code: green: >20 % activity*; blue: <20 % activity*; yellow: in general, poor activity; red: no activity
*For the color code activity is defined as the activity observed with carba-NADP(H), divided by the activity observed with NADP(H), times 100 %.

Entry	E. C. number	Enzyme name / UniprotKB or Accession No.	Organism	U · g^{-1} · 1000 NADP (H)	U · g^{-1} · 1000 carba-NADP(H)	Substrate	Reaction	Enzyme Assay
1	1.1.1.1	*Pfu*ADH (*N*-His) [3] / O73949	*Pyrococcus furiosus*	2.1	3.2	Benzaldehyde	Phenylmethanol + NAD(P)$^+$ ⇄ Benzaldehyde + NAD(P)H	60 °C /KPi pH 7.5/ 10 mM substrate
2	1.1.1.1	*Gst*ADH (*N*-His) [4] / P42328	*Geobacillus stearothermophilus*	1.3	1.8	Benzaldehyde	Phenylmethanol + NAD(P)$^+$ ⇄ Benzaldehyde + NAD(P)H	45 °C/MES pH 6.5/ 10 mM substrate
3	1.1.1.1	*Sto*ADH (*N*-His) [5] / F9VMI9	*Sulfolobus tokodaii*	4.2	4.9	Benzaldehyde	Phenylmethanol + NAD(P)$^+$ ⇄ Benzaldehyde + NAD(P)H	45 °C/MES pH 6.5/ 10 mM substrate
4	1.1.1.1	*Hl*ADH1 (Sigma)[6] / P00327	*Equus ferus caballus*	0.14	0.1	Butanal	Butanol + NAD$^+$ ⇄ Butanal + NADH	30 °C/Tris-HCl pH 8/ 5 mM substrate
5	1.1.1.1	*Ec*ADHZ2 [7] / W8T2B0	*Escherichia coli*	18	15	Butanal	Butanol + NADP$^+$ ⇄ Butanal + NADPH	30 °C/Tris-HCl pH 8/ 5 mM substrate
6	1.1.1.1	*Ec*ADHZ3 [7] / P27250	*Escherichia coli*	348	197	Butanal	Butanol + NADP$^+$ ⇄ Butanal + NADPH	30 °C/Tris-HCl pH 8/ 5 mM substrate
7	1.1.1.1	ADHZ3 variant LND[8]	*Escherichia coli*	174	190	"	"	"
8	1.1.1.1	ADHZ3 variant DIN[8]	*Escherichia coli*	174	0.1	"	"	"
9	1.1.1.1	*At*ADH1 / F7U804	*Agrobacterium tumefaciens*	1.5	1.3	Butanal	Butanol + NADP$^+$ ⇄ Butanal + NADH	30 °C/Tris-HCl pH 8/ 5 mM substrate
10	1.1.1.1	*Sc*ADH2 / P00331	*Saccharomyces cerevisiae*	0.07	0.05	Butanal	Butanol + NAD$^+$ ⇄ Butanal + NADH	30 °C/Tris-HCl pH 8/ 5 mM substrate
11	1.1.1.1	*Ssp*ADH / A0A075B5H4	*Sphingomonas spec.*	0.01	0.01	Butanal	Butanol + NADP$^+$ ⇄ Butanal + NADPH	30 °C/Tris-HCl pH 8/ 5 mM substrate
12	1.1.1.27	LDH (Carl Roth) [9]	Rabbit muscle	17.2	2.3	Pyruvat	(*S*)-lactate + NAD$^+$ ⇄ pyruvate + NADH	30 °C/Tris-HCl pH 8/ 5 mM substrate
13	1.1.1.46	*Bm*Arabinose DH (*N*-His) / A4JKL5	*Burkholderia multivorans*	8.0	2.0	L-Arabinose	L-arabinose + NADP$^+$ ⇄ L-arabinono-1,4-lactone + NADPH + H+	30 °C/Tris-HCl pH 8.0/ 0.5 mM cofactor / 400 mM substrate
14	1.1.1.46	*Bm*Arabinose DH (*C*-His) / A4JKL5	"	13	10	"	"	"

15	1.1.1.47	SsGlucose DH (*C*-His)[10] / O93715	*Sulfolobus solfactorius*	3.3	1.4	**D-Glucose**, D-Xylose, D-Galactose, L-Arabinose	D-Glucose + NAD(P) $^+$ ⇄ Glucono-δ-lactone + NAD(P)H	45 °C - (65 °C) /Tris-HCl pH 8/ 5 mM substrate
16	1.1.1.47	*Bs*Glucose DH $_{E170K\_Q252L}$ (*N*-His) [11] / Q9F5L5	*Bacillus subtilis*	1100	120	D-Glucose	D-Glucose + NAD(P)$^+$ ⇄ Glucono-δ-lactone + NAD(P)H	45 °C/Tris-HCl pH 7.5/ 500 mM substrate
17	1.1.1.49	*Sc*Glucose-6-Phosphate DH / P11412	*Saccharomyces cerevisiae*	245	14	Glucose-6-Phosphate	D-glucose 6-phosphate + NADP$^+$ ⇄ 6-phospho-D-glucono-1,5-lactone + NADPH	30 °C/Tris-HCl pH 8/ 5 mM substrate
18	1.1.1.49	*Lm*Glucose-6-Phosphate DH [12] / P11411	*Leuconostoc mesenteroides*	78	35	Glucose-6-Phosphate	D-glucose 6-phosphate + NADP$^+$ ⇄ 6-phospho-D-glucono-1,5-lactone + NADPH	30 °C/Tris-HCl pH 8/ 5 mM substrate
19	1.1.1.203	*At*Uronate DH [13] / Q7CRQ0	*Agrobacterium tumefaciens*	1.0	0.13	D-Galacturonate / Glucuronic acid	Beta-D-galacturonate + NAD$^+$ ⇄ D-galactaro-1,5-lactone + NADH	30 °C/Tris-HCl pH 8/ 5 mM substrate
20	1.1.1.203	*Sv*Uronate DH [13] / ZP_07302919.1	*Streptomyces viridochromogenes*	0.2	0.05	D-Galacturonate/ Glucuronic acid	Beta-D-galacturonate + NAD$^+$ ⇄ D-galactaro-1,5-lactone + NADH	30 °C/Tris-HCl pH 8/ 5 mM substrate
21	1.2.1.3	*Ta*AlDH (*C*-His) [14]	*Thermoplasma acidophilum*	24	0,3	Glyceraldehyde	Glyceraldehyde + NAD(P)$^+$ ⇄ Glycerate + NAD(P)H	45 °C/Tris-HCl pH 8/ 10 mM substrate
22	1.2.1.3	*Ta*ALD $_{F34M/W271S/S405N}$[14]	"	1	0	"	"	"
23	1.2.1.3	*Bst*ALDH [15] / P42329	*Bacillus stearothermophilus*	1.8	1.2	Hexanal	Hexanal + NAD(P)$^+$ ⇄ Hexanoic acid + NAD(P)H + H_2O	30 °C/Tris-HCl pH 8/ 5 mM substrate
24	1.2.1.24	*Ec*Succinic Semialdehyde DH / H4UMS0	*Escherichia coli*	150	2.5	Succinic Semialdehyde	succinic semialdehyde + NADP+ ⇄ succinate + NADPH	30 °C/ Tris-HCl pH 7.5 / 2.5 mM substrate
25	1.2.1.26	*α*-Ketoglutaric Semialdehyde DH [16] / Q88JR4	*Pseudomonas putida*	18.2	6.9	αKGSA	α-Ketoglutaric Semialdehyde + NADP$^+$ ⇄ α-Ketoglutarate + NADPH	30 °C/Tris-HCl pH 8/ 5 mM KGSA (5 µL from 283 mM in-house synthesis)
26	1.4.1.3	L-Glutamic DH / P00366	*Bovine liver*	100	90	L-Glutamate	L-glutamate + NAD(P)+ ⇄ α-ketoglutarate + NAD(P)H + NH_4^+	30 °C/Tris-HCl pH 8/ 5 mM substrate
27	1.5.1.-	*Asp*RedAm (*N*-His) [17] / Q2TW47	*Aspergillus oryzae*	5.1*	4.8*	***Hexanal** + ***Cyclo-**, 3-Buten-, Methyl-, Isopropyl amine	carbonyl compounds + prim. amine + NADPH → reductive coupling + NADP$^+$	30 °C/Tris-HCl pH 7.5/ 1 mM substrate
28	1.5.1.-	*Ad*RedAm (*N*-His) [17] / C5GTJ9	*Blastomyces dermatitidis*	5.5*	7.4*	***Hexanal** + ***Cyclo-**, 3-Buten-, Methyl-, Isopropyl amine	carbonyl compounds + prim. amine + NADPH → reductive coupling + NADP$^+$	45 °C/Tris-HCl pH 7.5/ 1 mM substrate
29	1.5.1.48	IRED-(*S*)-*Pe* [18] / WP_010497949.1	*Paenibacillus elgii*	0.3	0.3	3,4-Dihydroisochinolin	3,4-Dihydroisochinolin + NADPH → 1,2,3,4-tetrahydroisoquinoline	30 °C/Tris-HCl pH 7.5/ 1 mM substrate
30	1.6.2.4	P450BM3 (CYP102A1) [19] / P14779	*Bacillus megaterium*	0.5	0.4	Stearic acid	Stearic acid + O_2 → Hydroxy stearic acid + H_2O	30 °C/Tris-HCl pH 8/ 0,5 mM Stearic acid (DMSO)
31	1.6.3.1	*Lp*Nox [20] / F6IVN5	*Lactobacillus pentosus*	4.5	4.0	(carba)-NADPH	NAD(P)H → NAD(P)$^+$ + H_2O	30 °C/Tris-HCl pH 8/ 1 mM (c)NADPH
32	1.6.99.1	*Ts*ER (CrS)[21] / B0JDW3	*Thermus scotoductus*	2.6	2.4	Cyclohexenone	Cyclohexenone + NAD(P)H ⇄ Cyclohexanone + NAD(P)$^+$	30 °C/Tris-HCl pH 8/ 5 mM substrate
33	1.14.13.22	CHMO (BVMO) [22] / P12015	*Acinetobacter sp.*	3.6	3.4	Cyclohexanone	cyclohexanone + NADPH + H^+ + O_2 → caprolactone + NADP$^+$ + H_2O	30 °C/ Tris-HCl pH 8.0 / 2 mM substrate
34	1.14.13.84	HAPMO (BVMO) [23] / Q88J44	*Pseudomonas putida* JD1	7.5	7.0	(4-hydroxyphenyl)ethan-1-one	(4-hydroxyphenyl)ethan-1-one + NADPH + H^+ + O_2 → 4-hydroxyphenyl acetate + NADP$^+$ + H_2O	30 °C/ Tris-HCl pH 8.0 / 2 mM substrate
35	1.20.1.1	*Pp*PTDH / Q88HI1	*Pseudomonas putida*	1.4	0.3	Phosphite (Phosphonate)	Phosphite + NADP+ ⇄ Phosphate + NADPH	30 °C/ pH 6.5/ 100 mM substrate

36	1.20.1.1	*Ps*PTDH [24] Q137R/I150F/Q215L/R275Q/L276Q/A319E/ V315A/Q132R/V71I/E130K/I313L/A325V / O69054	*Pseudomonas stutzeri*	17.2	2	Phosphite (Phosphonate)	Phosphite + NADP+ ⇄ Phosphate + NADPH	45 °C/ pH 6.5/ 100 mM substrate
37	1.1.1.27	*Ec*AldA (*Ec*LAlDH) / P25553	*Escherichia coli*	0	0	(*S*)-Lactaldehyde / Isobutyraldehyde	(*S*)-Lactaldehyde + NAD^+ + H_2O ⇄ (*S*)-Lactate + NADH	30 °C/Tris-HCl pH 8/ 5 mM substrate
38	1.1.1.27	*Bst*ALDH [15]	*Bacillus stearothermophilus*	0	0	Glyceraldeyhde-3-Phosphate	Glyceraldehyde-3-Phosphate NAD^+ ⇄ 1,3-Bisphosphoglycerat + NADH	30 °C/Tris-HCl pH 8/ 5 mM substrate
39	1.1.1.30	*Rs* 3-Hydroxybutyrate DH / A4WSN7	*Rhodopseudomonas spheroides*	0.72	0.01	(*R*)-3-Hydroxybutyrate	(*R*)-3-Hydroxybutyrate + $NAD(P)^+$ ⇄ Acetoacetat + NADP(H)	30 °C/Tris-HCl pH 8/ 5 mM substrate
40	1.1.1.45	*Rl*L-Gulonate 3 DH	*Rabbit muscle*	0	0	(*S*)-3-Hydroxybutyrate	L-Gulonate + NAD^+ ⇄ 3-dehydro-L-gulonate + NADH + H+	30 °C/Tris-HCl pH 8/ 5 mM substrate
41	1.1.1.47	*Ta*Glucose DH[25] / P13203	*Thermoplasma acidophilum*	0.02	0	D-Glucose	D-Glucose + NAD^+ ⇄ Glucono-δ-lactone + NADH	45 °C/Tris-HCl pH 8/ 10 mM substrate
42	1.1.1.163	*Sm*Meso 2,3-Butanediol DH [26]	*Bacillus cereus*	0	0	Meso 2,3-Butanediol	(2*R*,3*S*)-Butane-2,3-diol + NAD^+ ⇄ Acetoin + NADH	30 °C/Tris-HCl pH 8/ 5 mM substrate
43	1.1.1.198	Borneol DH	*Pseudomonas sp.*	0.05	0.05	(+)-Borneol	(+)-Borneol + NAD^+ ⇄ (+)-Camphor + NADH + H^+	30 °C/ Tris-HCl pH 7.5 / 2.5 mM substrate
44	1.1.1.203	*Og*Uronate DH [13]	*Oceanicola granulosus*	0	0	D-Galacturonate/ Glucuronic acid	*β*-D-galacturonate + NAD^+ ⇄ D-galactaro-1,5-lactone + NADH	30 °C/Tris-HCl pH 8/ 5 mM substrate
45	1.2.1.2	Formate DH / O13437	*Candida boidinii*	0	0.01	Formate	Formate + NAD^+ ⇄ CO_2 + NADH	30 °C/KP_i pH 7.5/ 100 mM substrate
46	1.2.1.3	*Ec*AldX (*Ec*ALDH)	*Escherichia coli*	0.1	0.05	Propionaldehyde	Propionaldehyde + NAD(P) ⇄ Propionic acid + NAD(P)H	30 °C/Tris-HCl pH 8/ 5 mM Propionaldehyde
47	1.2.1.3	*Nm*ALDH (AldA) / X5EN19	*Neisseria meningitidis*	0	0	Glyceraldeyhde-3-Phosphate	Glyceraldehyde-3-Phosphate NAD^+ ⇄ 1,3-Bisphosphoglycerat + NADH	30 °C/Tris-HCl pH 8/ 5 mM substrate
48	1.2.1.12	Glyceraldehyde-3-Phosphate DH / P46406	*Rabitt muscle*	0	0	Glyceraldehyde-3-Phosphate	Glyceraldehyde-3-Phosphate NAD^+ ⇄ 1,3-Bisphosphoglycerat + NADH	30 °C/Tris-HCl pH 8/ 5 mM substrate
49	1.4.1.1	*Bs*Alanine DH / Q08352	*Bacillus subtilis*	0.01	0	L-Alanine	L-Alanine + H_2O + NAD^+ ⇄ Pyruvate + NH_3 + NADH	30 °C/Tris-HCl pH 8/ 5 mM substrate
50	1.6.1.1	*Ec*Transhydrogenase [27] / P27306	*Escherichia coli*	2	0	NADH/ carba-$NADP^+$	NADPH + (thio)NAD^+ ⇄ $NADP^+$ + (thio)NADH	30 °C/Tris-HCl pH 8/ 5 mM NADH + 5 mM carba-$NADP^+$
51	1.6.2.5	*Pp*XylG [28] / P23105	*Pseudomonas putida*	0	0	o-Phthaldialdehyde / Benzaldehyde	2-hydroxymuconate-6-semialdehyde + NAD^+ + H_2O ⇄ (2Z,4E)-2-hydroxyhexa-2,4-dienedioate + NADH	25 °C/KPi pH 9.5/ 10 mM substrate
52	1.8.1.4	*Ck*Diaphorase[29] / A5MZI4	*Chlostridium kluyveri*	0.02	0	Methylene blue	Methylene blue reduction	30 °C/ according to diaphorase assay[1]

Kinetic Measurements

Plots of kinetic measurements for cofactor and substrate of different enzymes were generated for better understanding of carba-NADP+ binding.

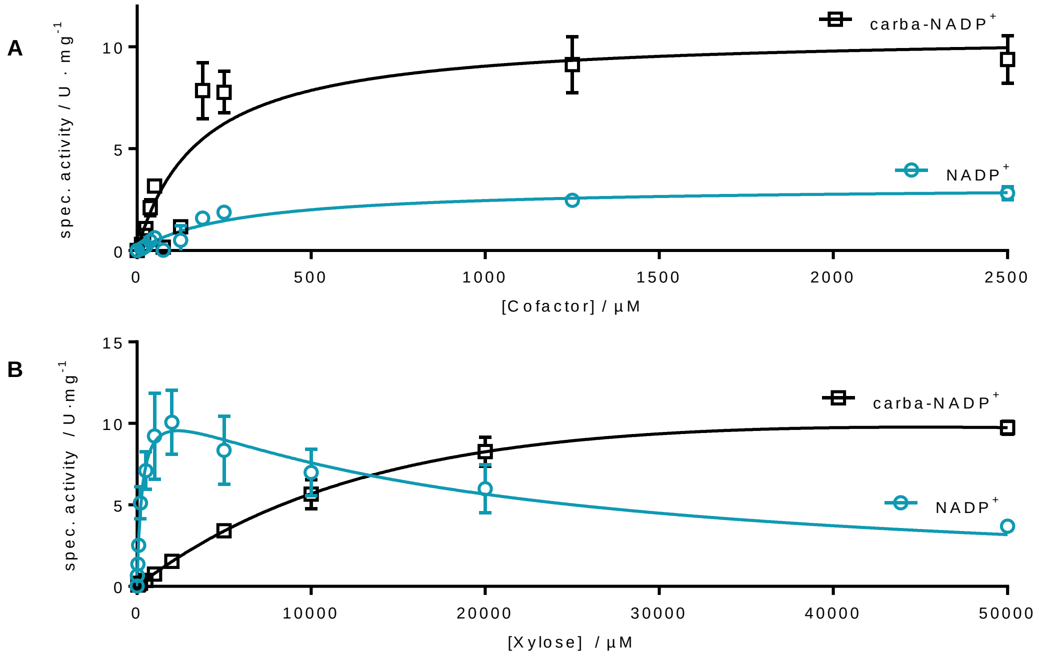

Figure S3 Kinetic measurement of glucose dehydrogenase from *Saccharolobus solfataricus* varying the concentration of **(A)** cofactors NADP+ (blue circles o), carba-NADP+ (black squares □) with a constant substrate concentration (50 mM) and **(B)** D-xylose with constant cofactor concentration of NADP+ or carba-NADP+ (2.5 mM). Reaction conditions: 65 °C, 100 mM Tris HCl pH 7.5, 0.095 mg enzyme, 0.2 mL reaction volume.

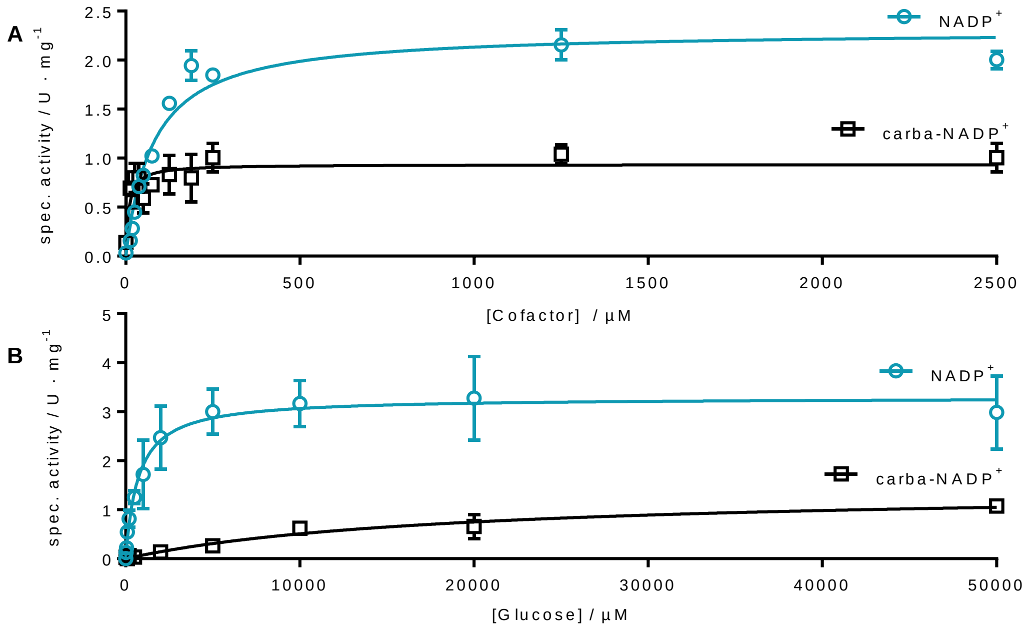

Figure S4 Kinetic measurement of glucose dehydrogenase from *Saccharolobus solfataricus* varying the concentration of **(A)** cofactors NADP+ (blue circles o), carba-NADP+ (black squares □) with a constant substrate concentration (50 mM) and **(B)** D-glucose with constant cofactor concentration of NADP+ or carba-NADP+ (2.5 mM). Reaction conditions: 65 °C, 100 mM Tris HCl pH 7.5, 0.095 mg enzyme, 0.2 mL reaction volume.

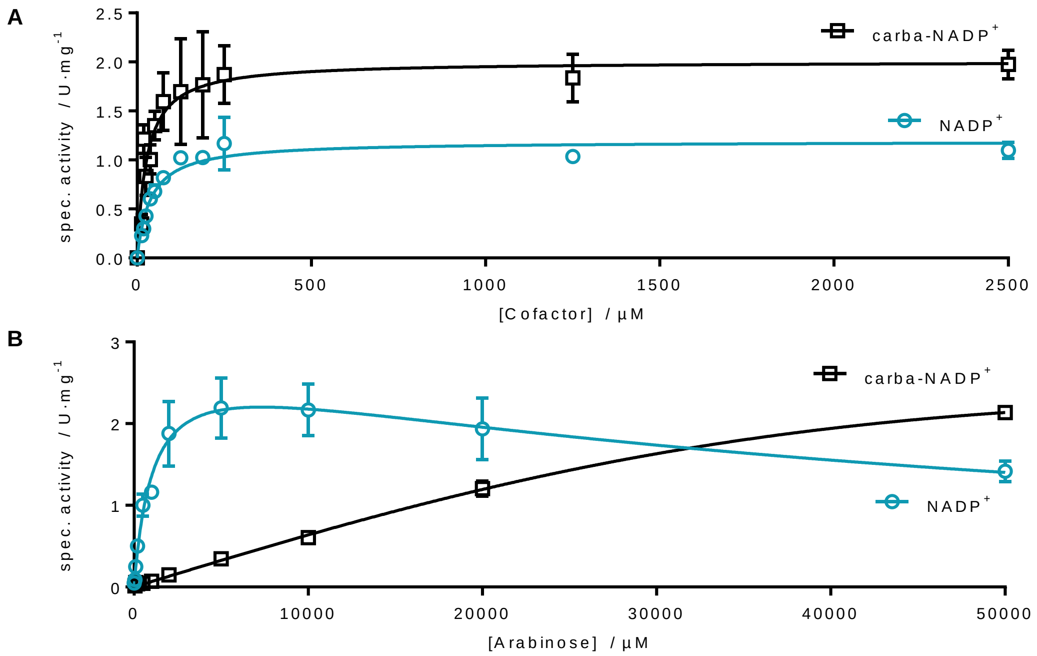

Figure S5 Kinetic measurement of glucose dehydrogenase from *Saccharolobus solfataricus* varying the concentration of **(A)** cofactors $NADP^+$ (blue circles o), carba-$NADP^+$ (black squares □) with a constant substrate concentration (50 mM) and **(B)** L-arabinose with constant cofactor concentration of $NADP^+$ or carba-$NADP^+$ (2.5 mM). Reaction conditions: 65 °C, 100 mM Tris HCl pH 7.5, 0.095 mg enzyme, 0.2 mL reaction volume.

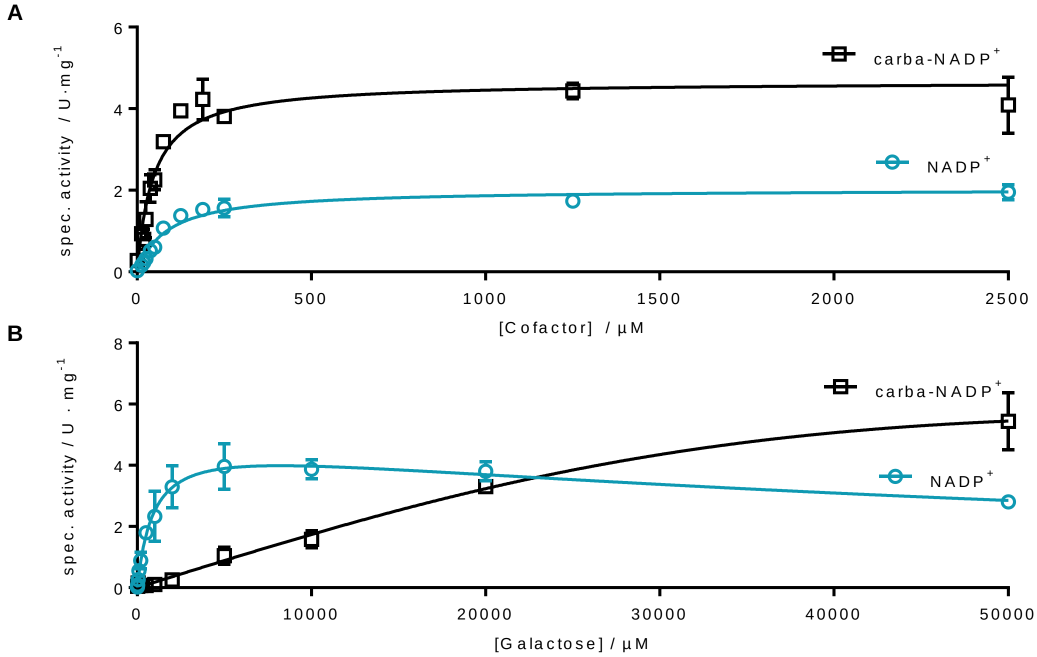

Figure S6 Kinetic measurement of glucose dehydrogenase from *Saccharolobus solfataricus* varying the concentration of **(A)** cofactors $NADP^+$ (blue circles o), carba-$NADP^+$ (black squares □) with a constant substrate concentration (50 mM) and **(B)** D-galactose with constant cofactor concentration of $NADP^+$ or carba-$NADP^+$ (2.5 mM). Reaction conditions: 65 °C, 100 mM Tris HCl pH 7.5, 0.095 mg enzyme, 0.2 mL reaction volume.

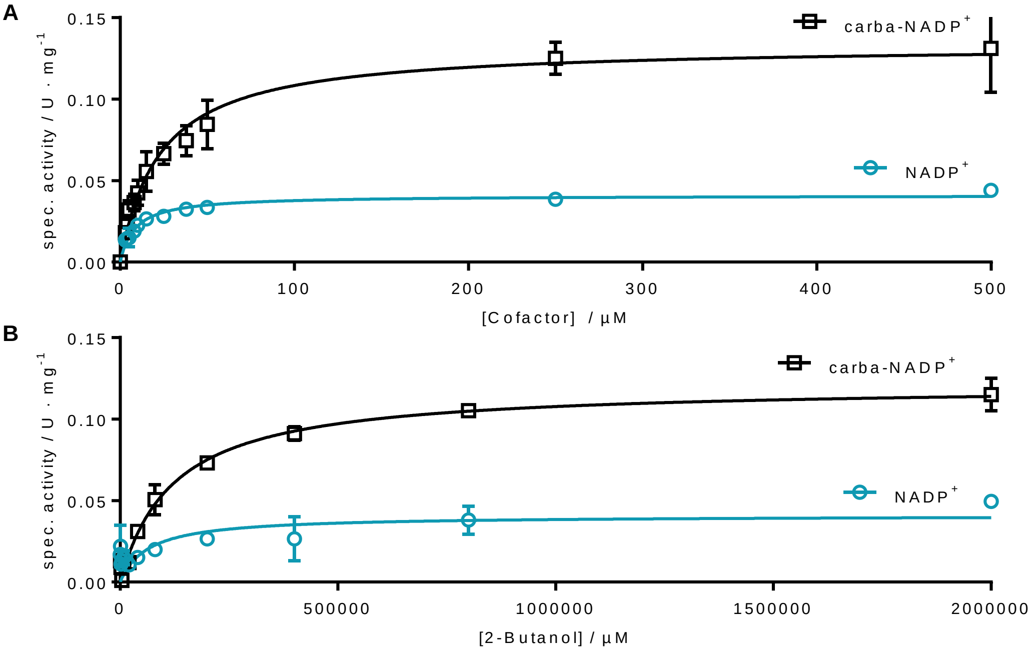

Figure S7 Kinetic measurement of alcohol dehydrogenase from *Pyrococcus furiosus* varying the concentration of **(A)** cofactors NADP+ (blue circles o), carba-NADP+ (black squares □) and with a constant substrate concentration (2 M) **(B)** 2-butanol with constant cofactor concentration of NADP+ or carba-NADP+ (0.5 mM). Reaction conditions: 65 °C, 33 mM Gly-NaOH pH 10.5, 0.163 mg enzyme, 0.2 mL reaction volume.

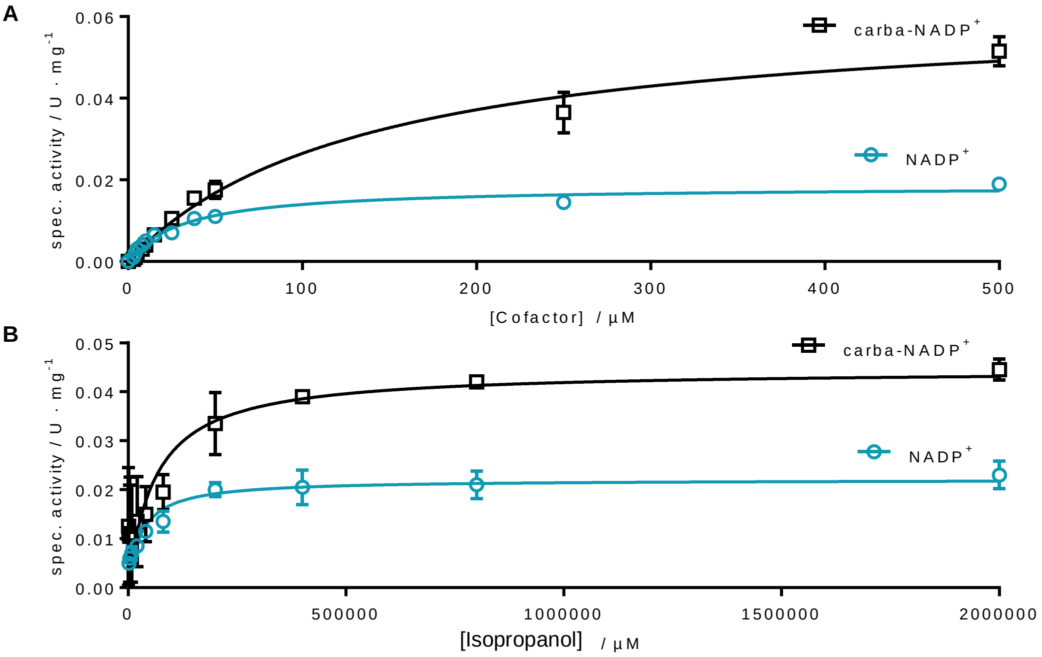

Figure S8 Kinetic measurement of alcohol dehydrogenase from *Pyrococcus furiosus* varying the concentration of **(A)** cofactors NADP+ (blue circles o), carba-NADP+ (black squares □) with a constant substrate concentration (2 M) and **(B)** isopropanol with constant cofactor concentration of NADP+ or carba-NADP+ (0.5 mM). Reaction conditions: 65 °C, 33 mM Gly-NaOH pH 10.5, 0.163 mg enzyme, 0.2 mL reaction volume.

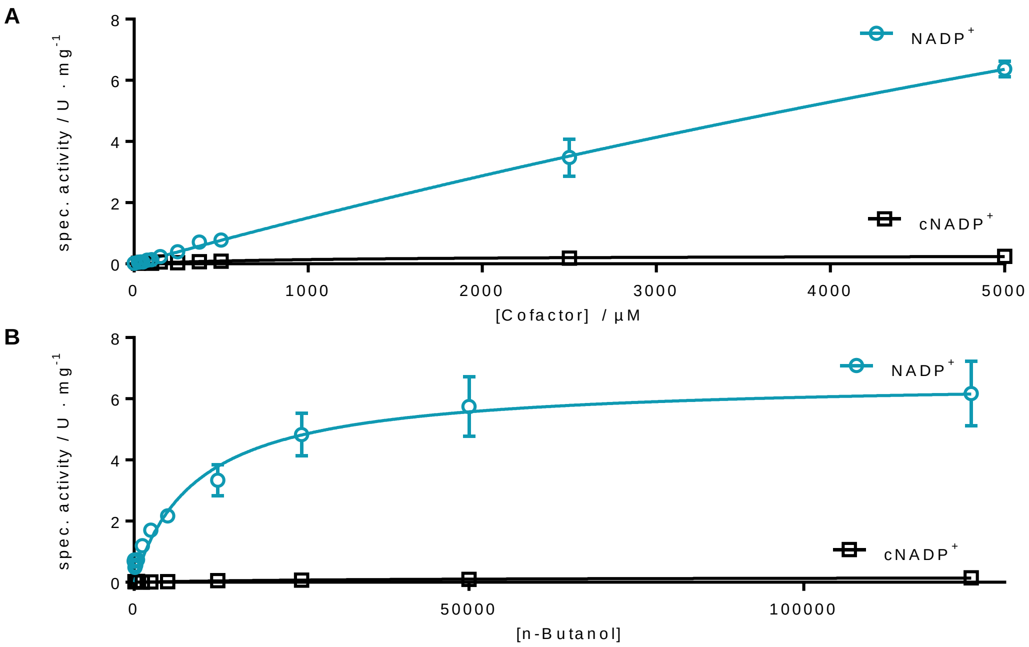

Figure S9Kinetic measurement of alcohol dehydrogenase from *Geobacillus stearothermophilus* varying the concentration of **(A)** cofactors NADP$^+$ (blue circles o), carba-NADP$^+$ (black squares □) with a constant substrate concentration (125 mM) and **(B)** *n*-butanol with constant cofactor concentration of NADP$^+$ or carba-NADP$^+$ (5 mM). Reaction conditions: 65 °C, 100 mM Tris-HCl pH 7.8, 0.0082 mg enzyme, 0.2 mL reaction volume.

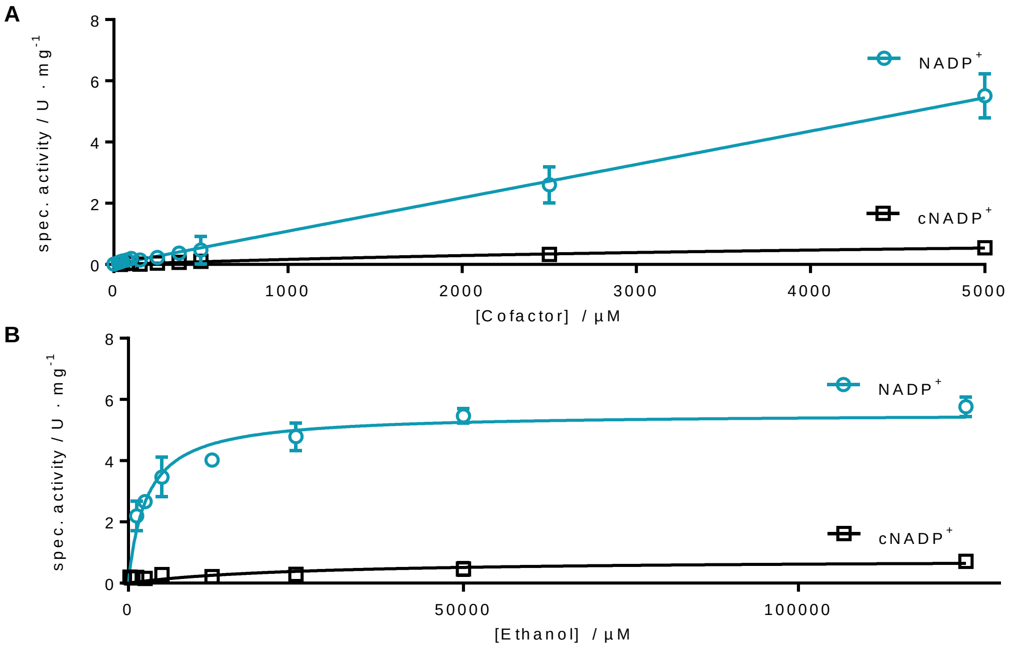

Figure S10 Kinetic measurement of alcohol dehydrogenase from *Geobacillus stearothermophilus* varying the concentration of **(A)** cofactors NADP$^+$ (blue circles o), carba-NADP$^+$ (black squares □) with a constant substrate concentration (125 mM) and **(B)** ethanol with constant cofactor concentration of NADP$^+$ or carba-NADP$^+$ (5 mM). Reaction conditions: 65 °C, 100 mM Tris-HCl pH 7.8, 0.0082 mg enzyme, 0.2 mL reaction volume.

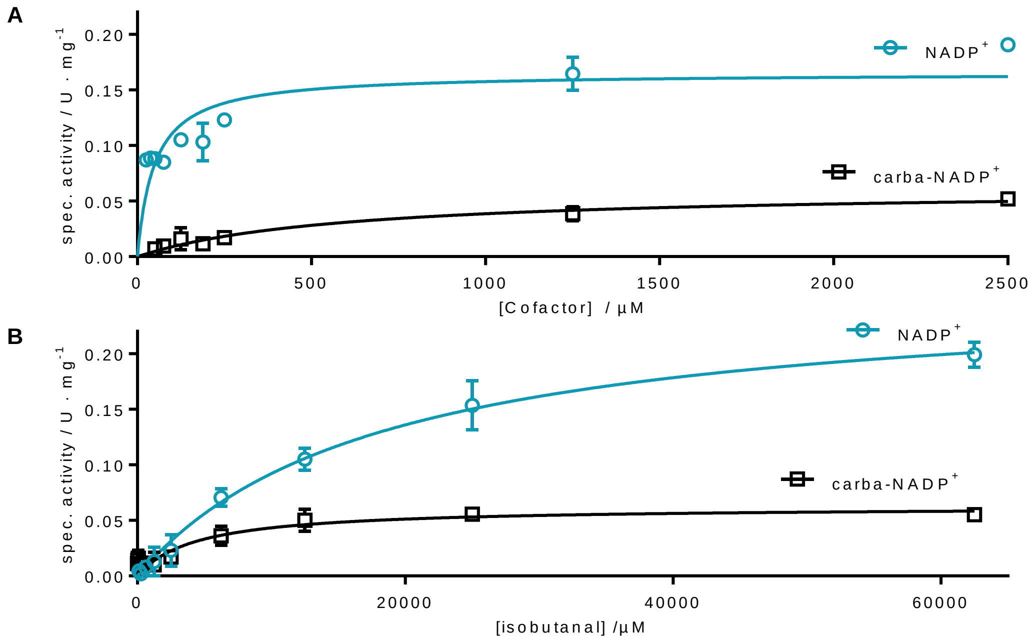

Figure S11 Kinetic measurement of aldehyde dehydrogenase from *Geobacillus stearothermophilus* varying the concentration of **(A)** cofactors NADP+ (blue circles o), carba-NADP+ (black squares □) with a constant substrate concentration (62.5 mM) and **(B)** butanal with constant cofactor concentration of NADP+ or carba-NADP+ (2.5 mM). Reaction conditions: 65 °C, 100 mM Tris-HCl pH 7.8, 0.0082 mg enzyme, 0.2 mL reaction volume.

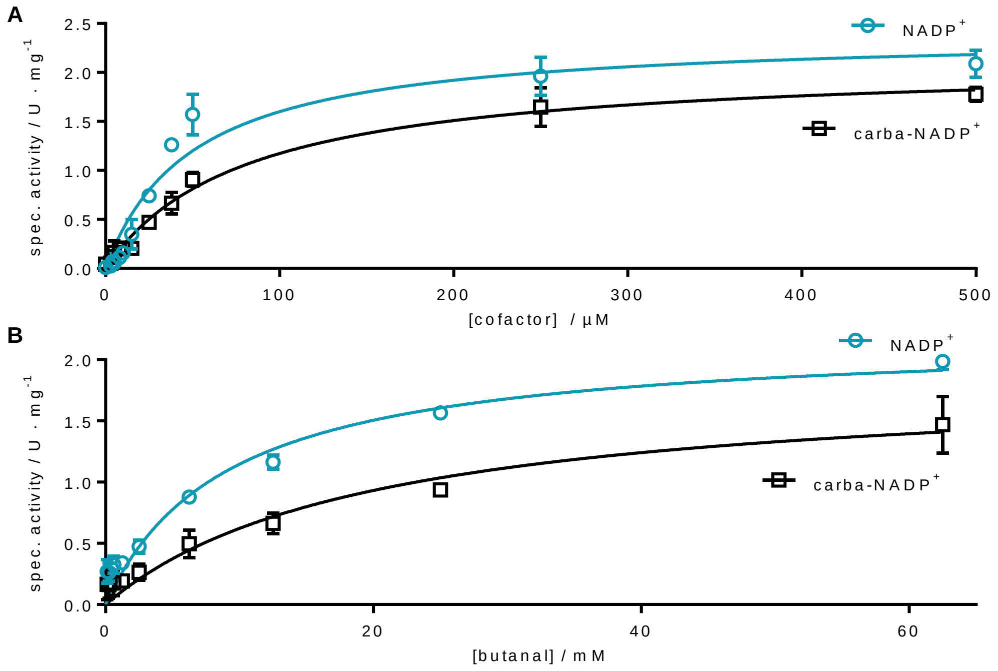

Figure S12 Kinetic measurement of aldehyde dehydrogenase from *Geobacillus stearothermophilus* varying the concentration of **(A)** cofactors NADP+ (blue circles o), carba-NADP+ (black squares □) with a constant substrate concentration (62.5 mM) and **(B)** butanal with constant cofactor concentration of NADP+ or carba-NADP+ (2.5 mM). Reaction conditions: 65 °C, 100 mM Tris-HCl pH 7.8, 0.0082 mg enzyme, 0.2 mL reaction volume.

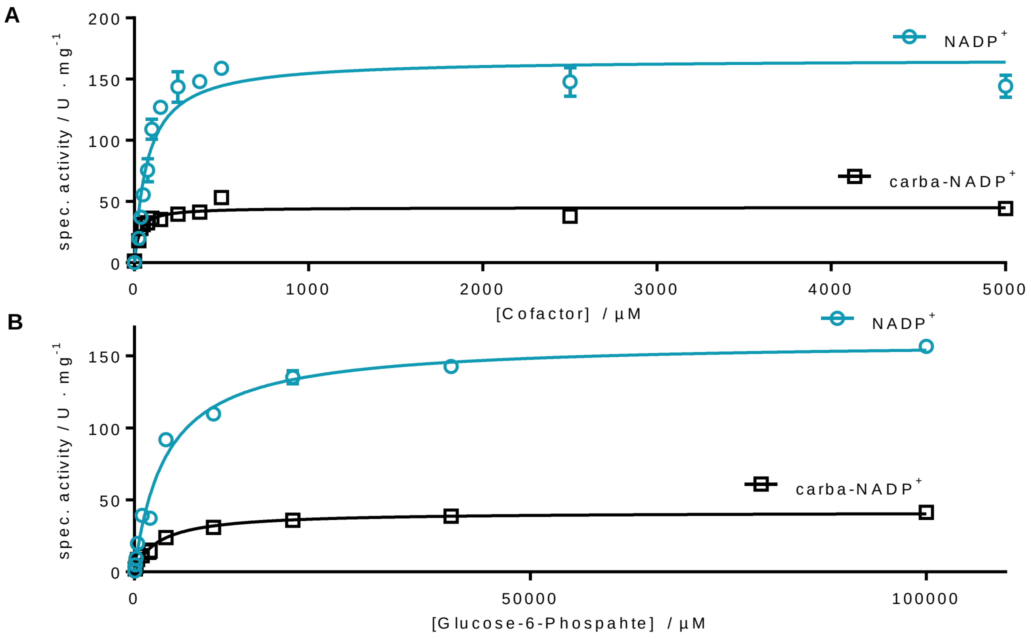

Figure S13 Kinetic measurement of glucose-6-phosphate dehydrogenase from *Saccharomyces cerevisiae* varying the concentration of **(A)** cofactors NADP+ (blue circles o), carba-NADP+ (black squares □) with a constant substrate concentration (100 mM) and **(B)** glucose-6-phosphate with constant cofactor concentration of NADP+ or carba-NADP+ (2.5 mM). Reaction conditions: 30 °C, 100 mM Tris HCl pH 7.5, 0.00125 mg enzyme, 0.2 mL reaction volume.

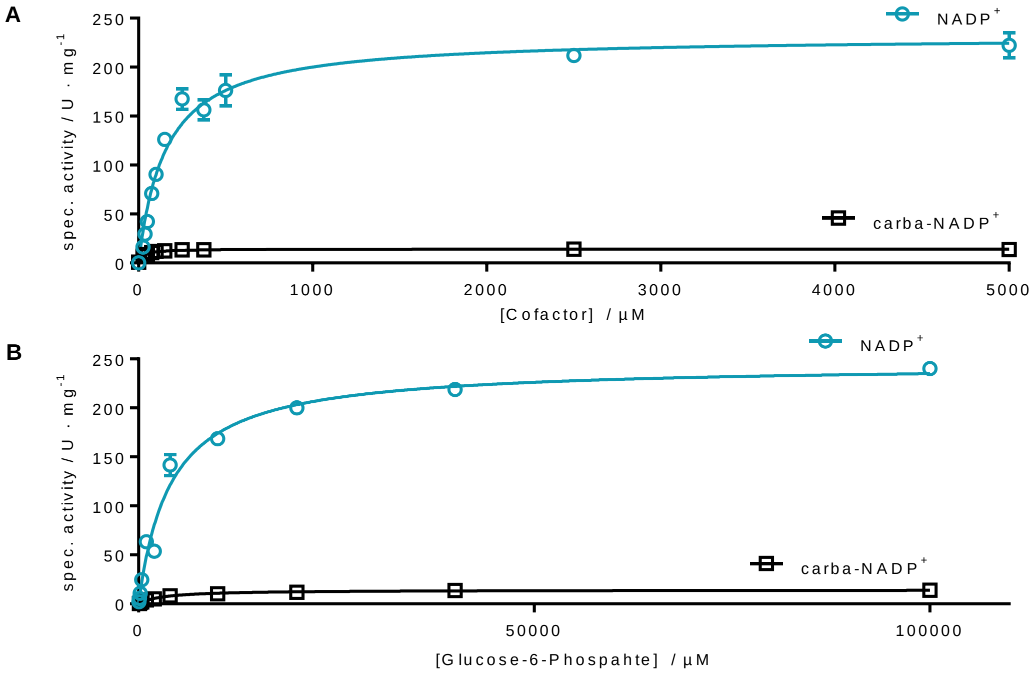

Figure S14 Kinetic measurement of glucose-6-phosphate dehydrogenase from *Leuconostoc mesenteroides* varying the concentration of **(A)** cofactors NADP+ (blue circles o), carba-NADP+ (black squares □) with a constant substrate concentration (100 mM) and **(B)** glucose-6-phosphate with constant cofactor concentration of NADP+ or carba-NADP+ (2.5 mM). Reaction conditions: 30 °C, 100 mM Tris HCl pH 7.5, 0.00125 mg enzyme, 0.2 mL reaction volume.

Comparison of kinetic constants

In the following, kinetic constants of the measurements presented above were extracted and summarized in a table (Table S5) for better comparison. For *Bst*ADH and the substrate ethanol no saturation could be determined for the cofactor.

Table S5 Kinetic constants for substrates and cofactors ($NADP^+$, carba-$NADP^+$) of 6 enzymes

Enzyme		*Ss*GDH				*Pfu*ADH		*Bst*ADH		*Bst*ALDH		G6PDH	
												Sc	*Lm*
Substrates		D-Glucose	D-Xylose	D-Galactose	L-Arabinose	2-Butanol	Isopropanol	*n*-Butanol	Ethanol	Butanal	Isobutanal	Glucose-6-Phosphate	
$NADP^+$	K_m Cofactor [µM]	80	290	86.7	38.2	8.5	31.8	20,790	no saturation detected	50.17	50	157	75
	K_m Substrate [µM]	725	305	940	1,090	64,295	30,205	9,345	2,739	9,120	18,190	4,050	3,980
	K_i Substrate [µM]	-	17,950	71,355	4,929	-	-	-	-	-	-	-	-
	V_{max} [$U \cdot mg^{-1}$]	3.3	3.2	2.0	2.8	0.04	0.02	32	5.5	2.4	0.17	245	166
	k_{cat} [s^{-1}]	2.25	2.18	1.4	1.9	0.03	0.01	19.4	3.3	2.1	0.1	234.9	150.3
	$k_{cat}/K_{m\,cof}$ [$mM^{-1} s^{-1}$]	28.09	7.51	15.71	49.92	3.25	0.43	0.93	-	42.19	3.00	1,496	2,004
	$k_{cat}/K_{m\,sub}$ [$mM^{-1} s^{-1}$]	38.75	24.64	16.71	45.79	0.05	0.01	0.10	-	4.63	0.16	369	503
carba-$NADP^+$	K_m Cofactor [µM]	10	180	47.2	27	23.0	135.5	1,060	no saturation detected	80.41	590	26	27
	K_m Substrate [µM]	18,110	12,205	52,940	65,380	34,440	61,140	36,910	255,600	19,850	4,430	3,100	3,015
	$K_{i\,Substrate}$ [µM]	-	-	-	-	-	-	-	-	-	-	-	-
	V_{max} [$U \cdot mg^{-1}$]	1.4	**12.5**	11.29	5	**0.130**	**0.06**	0,3	0.78	2.1	0.06	14	45
	k_{cat} [s^{-1}]	1.0	8.5	7.7	3.4	0.1	0.004	0.2	0.5	1.9	0.1	13.4	40.7
	$k_{cat}/K_{m\,cof}$ [$mM^{-1} s^{-1}$]	95.34	47.29	162.89	126.11	3.90	0.31	0.17	-	23.03	0.09	516	1,509
	$k_{cat}/K_{m\,sub}$ [$mM^{-1} s^{-1}$]	5.26	3.87	3.08	1.93	0.11	0.005	0.005	-	1.16	0.02	166	500

Spectral analysis of (carba)-NADPH

Owing to the carbocylic sugar, carba-NADPH has a shift of 18 - 20 nm in absorbance. The absorption maximum is thus 358 nm - 360 nm instead of 340 nm. Using the NAD(P)H Oxidase from *Lactobacillus pentosus* cofactors were checked if they were still intact from different experiments are still intact for biocatalytic conversion.

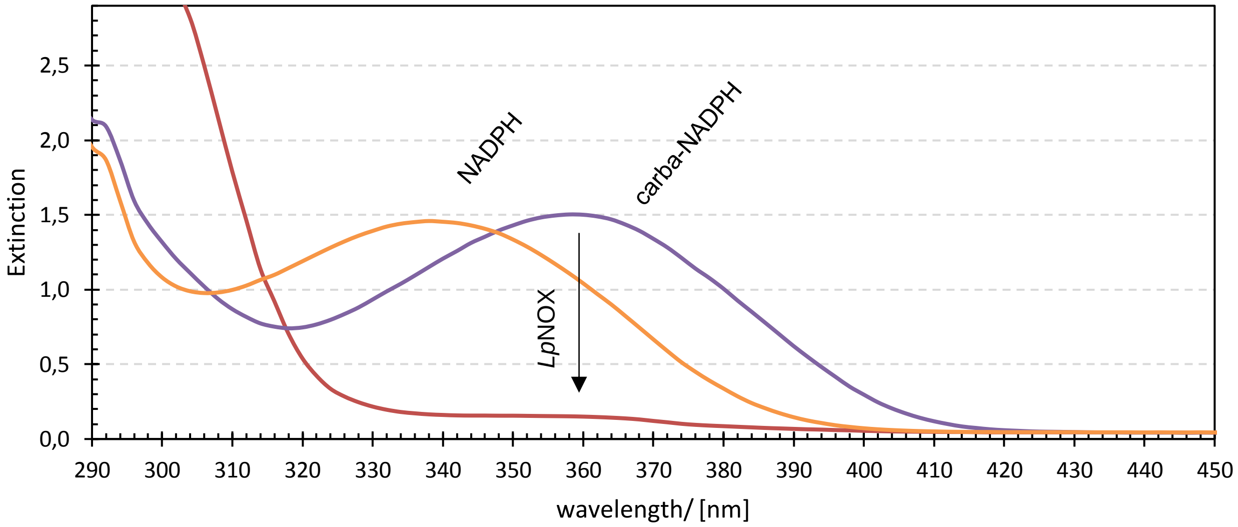

Figure S15 Spectral scanning of reduced nicotinamide cofactors (l.) NADPH (r.) carba-NADPH each 700 µM produced during glucose dehydrogenase reactions. Maximal absorption occurs at 340 nm for NADPH and 358 nm - 360 nm for carba-NADPH. After treatment with NAD(P)H Oxidase from *Lactobacillus pentosus* (*Lp*NOX) carba-NADPH is oxidized accompanied by almost complete loss of absorption at 360 nm. Increase at 280 nm is caused by the addition of additional enzyme.

3D modeling of nicotinamide cofactors

In silico studies of both nicotinamide cofactors (in the oxidized and in the reduced state) revealed different energetic minima. The rotamers of the nicotinamide moiety and the adenine group, in particular, are altered, which can affect enzyme-ligand interactions. Depending on the exact architecture of the enzyme's catalytic center, these changes can affect catalytic parameters.

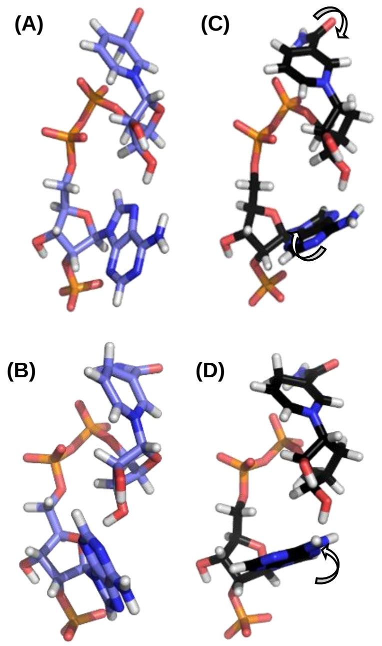

Figure S16 *In silico* minimization of **(A)** NADP **(B)** NADPH **(C)** carba-NADP$^+$ and **(D)** carba-NADPH shows different rotamers depending on the oxidation state and presence of the ribose oxygen. Structures were created with Chem3D and minimized several times in YASARA using the NOVA force field. Structures were rendered with PyMOL.

G6PDH structural differences

A quick comparative view of the aligned active sites reveals that the adenine binding region of the more active enzyme allows more conformations, due to an enlarged pocket; V86 in *Lm*G6PDH instead of Y95 in *Sc*G6PDH. Therefore, the energetically lower state having the twisted adenine moiety is not favored in *Sc*G6PDH. It should be mentioned that the overall sequence identity is only 35.08 % and other effects are also sure to affect ligand binding.

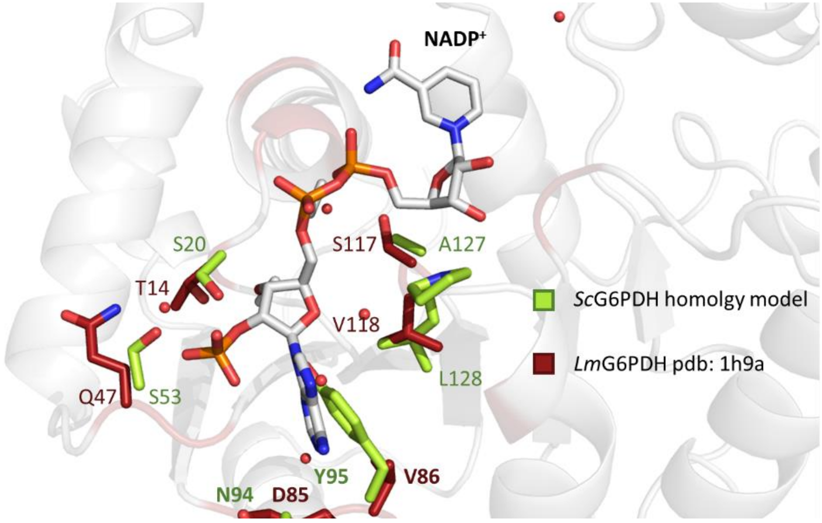

Figure S17 Aligned binding sites of glucose-6-phosphate dehydrogenases from *Saccharomyces cerevisiae* (homology model on 6e07.1.A) and *Leuconostoc mesenteroides* (pdb: 1h9a). All differences within 4 Å of the cofactor are shown as sticks.

References

[1] F. W. Studier, *Protein Expr. Purif.* **2005**, *41*, 207.
[2] H. Duefel, D. Heindl, C. Horn, T. Meier, R. Schmuck, WO 2011/012270 A1, **2011**.
[3] J. van der Oost, W. G. B. Voorhorst, S. W. M. Kengen, A. C. M. Geerling, V. Wittenhorst, Y. Gueguen, W. M. de Vos, *Eur. J. Biochem.* **2001**, *268*, 3062.
[4] E.-J. Shim, S.-H. Jeon, K.-H. Kong, *J. Microbiol. Biotechnol.* **2003**, *13*, 738.
[5] H. Yanai, K. Doi, T. Ohshima, *Appl. Environ. Microbiol.* **2009**, *75*, 1758.
[6] V. P. Fernandez, H. R. Mahler, V. J. Shiner Jr, *Biochemistry* **1962**, *1*, 259.
[7] A. Pick, B. Rühmann, J. Schmid, V. Sieber, *Appl. Microbiol. Biotechnol.* **2013**, *97*, 5815.
[8] A. Pick, W. Ott, T. Howe, J. Schmid, V. Sieber, *J. Biotechnol.* **2014**, *189*, 157.
[9] R. D. Cahn, N. O. Kaplan, L. Levine, E. Zwilling, *Science* **1962**, *136*, 962.
[10] P. Giardina, M. G. de Biasi, M. de Rosa, A. Gambacorta, V. Buonocore, *Biochem. J* **1986**, *239*, 517.
[11] E. Vázquez‐Figueroa, J. Chaparro‐Riggers, A. S. Bommarius, *ChemBioChem* **2007**, *8*, 2295.
[12] C. Olive, M. E. Geroch, H. R. Levy, *J. Biol. Chem.* **1971**, *246*, 2047.
[13] A. Pick, J. Schmid, V. Sieber, *Microb. Biotechnol.* **2015**, *8*, 633.
[14] F. Steffler, J.-K. Guterl, V. Sieber, *Enzyme and microbial technology* **2013**, *53*, 307.
[15] T. Imanaka, T. Ohta, H. Sakoda, N. Widhyastuti, M. Matsuoka, *J. Ferment. Bioeng.* **1993**, *76*, 161.
[16] Barbara Beer, *Dissertation: Development of enzymatic cascade reactions toward the synthesis of 1,4-butanediol*. Dissertation, **2018**.
[17] G. A. Aleku, S. P. France, H. Man, J. Mangas-Sanchez, S. L. Montgomery, M. Sharma, F. Leipold, S. Hussain, G. Grogan, N. J. Turner, *Nat. Chem.* **2017**, *9*, 961.
[18] M. Lenz, P. N. Scheller, S. M. Richter, B. Hauer, B. M. Nestl, *Protein Expr. Purif.* **2017**, *133*, 199.
[19] Y. Miura, A. J. Fulco, *J. Biol. Chem.* **1974**, *249*, 1880.
[20] C. Nowak, B. C. Beer, A. Pick, T. Roth, P. Lommes, V. Sieber, *Front. Microbiol.* **2015**, *6*, 957.
[21] D. J. Opperman, L. A. Piater, E. van Heerden, *J. Bacteriol.* **2008**, *190*, 3076.
[22] S. S. Barclay, J. M. Woodley, M. D. Lilly, P. L. Spargo, A. J. Pettman, *Biotechnol. Lett* **2001**, *23*, 385.
[23] J. Rehdorf, C. L. Zimmer, U. T. Bornscheuer, *Appl. Environ. Microbiol.* **2009**, *75*, 3106.
[24] T. W. Johannes, R. D. Woodyer, H. Zhao, *Appl. Environ. Microbiol.* **2005**, *71*, 5728.
[25] J. R. Bright, D. Byrom, M. J. Danson, D. W. Hough, P. Towner, *Eur. J. Biochem.* **1993**, *211*, 549.
[26] H. Hohagen, D. Schwarz, G. Schenk, L. W. Guddat, D. Schieder, J. Carsten, V. Sieber, *Bioresour. Technol.* **2017**, *245*, 1084.
[27] B. Boonstra, C. E. French, I. Wainwright, N. C. Bruce, *J. Bacteriol.* **1999**, *181*, 1030.
[28] M. I. Steele, D. Lorenz, K. Hatter, A. Park, Sokatch, JR, *J. Biol. Chem.* **1992**, *267*, 13585.
[29] S. Chakraborty, M. Sakka, T. Kimura, K. Sakka, *Biosci. Biotechnol. Biochem.* **2008**, *72*, 982.

Author Contributions

Ioannis Zachos performed all enzymatic experiments, studies on different cofactor stabilities and *in silico* calculations. Manuel Döring developed a semi-automated enzyme kinetic protocol using a liquid handling station and assisted in kinetic measurements. Georg Tafertshofer, Robert C. Simon supplied cofactor and performed experiments on thermal stability of cofactors. Volker Sieber supervised this study including ideas and revising the manuscript.
